# 5′ Rapid Amplification of cDNA Ends and Illumina MiSeq Reveals B Cell Receptor Features in Healthy Adults, Adults With Chronic HIV-1 Infection, Cord Blood, and Humanized Mice

**DOI:** 10.3389/fimmu.2018.00628

**Published:** 2018-03-26

**Authors:** Eric Waltari, Manxue Jia, Caroline S. Jiang, Hong Lu, Jing Huang, Cristina Fernandez, Andrés Finzi, Daniel E. Kaufmann, Martin Markowitz, Moriya Tsuji, Xueling Wu

**Affiliations:** ^1^Aaron Diamond AIDS Research Center, Affiliate of The Rockefeller University, New York, NY, United States; ^2^Hospital Biostatistics, The Rockefeller University, New York, NY, United States; ^3^Centre de Recherche du CHUM, Université de Montréal, Montreal, QC, Canada; ^4^Center for HIV/AIDS Vaccine Immunology and Immunogen Discovery (CHAVI-ID), La Jolla, CA, United States

**Keywords:** B cell repertoire, deep sequencing, HIV, cord blood, humanized mouse

## Abstract

Using 5′ rapid amplification of cDNA ends, Illumina MiSeq, and basic flow cytometry, we systematically analyzed the expressed B cell receptor (BCR) repertoire in 14 healthy adult PBMCs, 5 HIV-1+ adult PBMCs, 5 cord blood samples, and 3 HIS-CD4/B mice, examining the full-length variable region of μ, γ, α, κ, and λ chains for V-gene usage, somatic hypermutation (SHM), and CDR3 length. Adding to the known repertoire of healthy adults, Illumina MiSeq consistently detected small fractions of reads with high mutation frequencies including hypermutated μ reads, and reads with long CDR3s. Additionally, the less studied IgA repertoire displayed similar characteristics to that of IgG. Compared to healthy adults, the five HIV-1 chronically infected adults displayed elevated mutation frequencies for all μ, γ, α, κ, and λ chains examined and slightly longer CDR3 lengths for γ, α, and λ. To evaluate the reconstituted human BCR sequences in a humanized mouse model, we analyzed cord blood and HIS-CD4/B mice, which all lacked the typical SHM seen in the adult reference. Furthermore, MiSeq revealed identical unmutated IgM sequences derived from separate cell aliquots, thus for the first time demonstrating rare clonal members of unmutated IgM B cells by sequencing.

## Introduction

The B cell receptor (BCR) repertoire is immense due to V(D)J recombination and additional diversification mechanisms such as non-templated junction formation, class switching, and somatic hypermutation (SHM). Though B cells have been extensively characterized by cell surface marker staining (1), flow cytometric phenotyping has rarely been accompanied by BCR sequencing, which determines B cell diversity, antigenic specificity and binding affinity. Early Sanger sequencing (2, 3) provided limited numbers (hundreds to thousands) of BCR sequences but recent next-generation sequencing (NGS) provided up to 10^6^ reads per individual with 454 pyrosequencing and up to 10^7^ reads per individual with Ion Torrent and Illumina (4–6). In addition, 5′ rapid amplification of cDNA ends (RACE) was introduced to reduce PCR bias by incorporating a known 5′ oligo sequence in the reverse transcribed cDNA (7). With these advances, BCR sequencing and characterization has been applied to adult PBMCs (8–12), individual libraries (13, 14), HIV-1+ individuals (15–19), and adults before and after vaccination (20, 21), commonly reporting the BCR V-gene usage, SHM, and CDR3 length. However, these studies were often oriented to specific questions, limited to one to four individuals, covering only part of the BCR variable region, or γ chain only, and lacked phenotyping of the B cells sequenced. Therefore, characterization of general B cells and BCRs remains largely incomplete for healthy adults.

A more fully characterized reference BCR repertoire representing healthy adult PBMCs accompanied with basic immunophenotyping is urgently needed for comparisons with infectious diseases and other B cell-related conditions to identify potential changes at the BCR repertoire level. A BCR reference is also needed for comparisons with humanized mice that are frequently used to study the human immune system (HIS). Here, we examined the BCR repertoire of a HIS mouse model, the HIS-CD4/B mice (22), in which development of human CD4+ T cells and B cells is enabled by adeno-associated virus-based gene transfer (23) to transduce mice with genes encoding HLA class II and selected human cytokines. Because purified human CD34+ hematopoietic stem cells from fetal liver or cord blood samples were used to inject highly immunodeficient mice that lacked murine T and B cells, we included cord blood analysis for comparison.

The focal purpose of this study is to apply the state-of-art 5′ RACE PCR and Illumina 2 × 300 bp paired-end MiSeq platform to systematically examine full-length BCR variable region of μ, γ, α, κ, and λ chains with close to 10^7^ reads per individual to establish the reference BCR repertoires in healthy adults and cord blood samples. We then compared the repertoires of HIV-1+ subjects to the adult reference and compared the repertoires of HIS-CD4/B mice to cord blood. We aim to address three questions: (1) what is the reference V-gene usage, SHM, and CDR3 length in adult PBMCs, and how much variation exists among individuals? (2) How do BCR repertoires of HIV-1+ subjects compare to this reference? (3) How do BCR repertoires vary among HIS-CD4/B mice, cord blood cells, and adult PBMCs?

After antigenic activation and clonal expansion, clonal variants of the responded B cells are commonly found both by antigen-specific B cell sorting (24–26) and by NGS (27–29). However, before encountering an antigen, naïve B cells have not been clonally expanded, and thus replicates of naïve B cells are rare. Because of this rarity, it is almost impossible to find naïve B cell replicates generated during homeostatic expansion by single B cell sorting, yet it remains to be determined whether they could be identified by NGS, which covers hundreds of thousands to millions of sequencing reads. The available NGS data in this study allowed an initial attempt to address this question.

## Materials and Methods

### Human Samples, and Samples From Humanized Mice

Adult blood and cord blood samples were purchased from the New York Blood Center. Five HIV-1-infected PBMC samples (Table S1 in Supplementary Material) were obtained from two study cohorts: the Aaron Diamond AIDS Research Center Acute and Early HIV-1 Infection Cohort (30) and the Montreal Primary HIV-1 Infection Cohort (31, 32). All patient samples were collected under Institutional Review Board (IRB) approved protocols. The HIS-CD4/B mice have been previously reported (22, 23). Briefly, NOD.Cg-Prkdc^scid^ IL2rg^tmWjl^/Sz (NSG) mice were purchased from The Jackson Laboratories and maintained under specific pathogen-free conditions in the animal facilities at the Comparative Bioscience Center of The Rockefeller University. All animal experiments were carried out in strict accordance with the Policy on Humane Care and Use of Laboratory Animals of the United States Public Health Service. The protocol was approved by the Institutional Animal Care and Use Committee at The Rockefeller University (Assurance #A3081-01). Human fetal liver samples, from which CD34+ hematopoietic stem cells were purified, were obtained *via* a non-profit partner (Advanced Bioscience Resources, Alameda, CA, USA) without any information that would identify the subjects from whom the samples were derived. Therefore, IRB approval was not required for the use of these samples.

### Sample Preparation for 5′ RACE and Deep Sequencing

Blood donor PBMCs, cord blood cells, and HIS-CD4/B mouse splenocytes were isolated using Ficoll-Pacque, with ACK buffer lysing red blood cells. The cellular mRNA was extracted using the Oligotex Direct mRNA Mini Kit (Qiagen), eluted in 200 μl buffer, and concentrated to 10–25 μl using Ultra 0.5 ml Centrifugal filters (Amicon). For 5′ RACE cDNA synthesis, each 10 μl mRNA was mixed with 1 μl Oligo dT_12–18_ at 12 μM (Life Technologies) at 70°C for 1 min and then −20°C for 1 min, followed by addition of 1 μl SMARTer Oligo at 12 μM (Clontech), 4 μl 5× first-strand buffer, 1 μl DTT at 20 mM, 1 μl dNTP at 10 mM each, 1 μl RNaseOUT, and 1–3 μl SuperScript II (Life Technologies). The mixtures were incubated at 42°C for 2 h and then passed through a PCR cleanup spin column (Machery-Nagel).

To maximize the use of precious clinical specimens, we amplified the variable regions of μ, γ, α, κ, and λ chains together from a single cDNA template equivalent to transcripts from 3–5 million cells, using the KAPA HiFi qPCR kit (KAPA Biosystems) with a universal 5′ RACE primer IIA (Clontech), 5′AAGCAG TGGTATCAACGCAGAG 3′, and a mixture of gene-specific 3′ primers: 3′ Mu-R, 5′ ATTCTCACAGGA GACGAGGGGGAAAAGGGTTG 3′; 3′ Gamma-R, 5′ GGGGAAGACCGATGGGCCCTTGGTGGARG 3′; 3′ Alpha-R, 5′ CGGGAAGACCTTGGGGCTGGTCGG 3′; 3′ Kappa-R, 5′ GGAAGATGAAGACAGA TGGTGCAGCCACAG 3′, and 3′ Lambda-R, 5′ CCTTGTTGGCTTGRAGCTCCTCAGAGGAGG 3′. Primers each contained a unique 8 bp Illumina barcode for demultiplexing after Miseq sequencing. For PBMCs, the PCR cycling conditions were 98°C for 45 s, 16–22 cycles of 98°C for 15 s, 65°C for 30 s, and 72°C for 45 s, followed by 72°C for 3 min. For HIS-CD4/B mice and cord blood samples, 18–25 cycles were used due to fewer B cells in these samples. The PCR products were loaded on 2% E-gels (Life Technologies) for visualization and extraction, with a final buffer exchange using the PCR Micro Kit (Life Technologies). The eluted PCR DNA was used for Illumina MiSeq library preparation and 2 × 300 bp paired-end indexed sequencing at the Rockefeller University Genomics Resource Center or the New York Genome Center, with two to three PCR samples multiplexed per run.

### Bioinformatics Analyses and Pipeline

The raw 2 × 300 paired-end reads from Illumina MiSeq were processed as follows:

(1) demultiplexing: the 8-bp Illumina barcodes were used to split reads by individual and by μ, γ, α, κ, and λ chains. To account for the imperfect initial incorporation of nucleotides during primer synthesis, we also included reads with partial barcode (minimum 4 bp) and the adjacent 4-bp primer sequence. In indexed library runs, reads with a minimum 8-bp primer sequence were also included.
(2) initial *IGBLAST* to identify BCR reads and join paired-ends: to remove non-BCR reads, we ran an initial in-house *IGBLAST*^1^ using a germline database of only functional V-genes from IMGT,^2^ rejecting reads with a bitscore <75 that indicates a match to less than 50 bp of BCR sequence. We also used the *IGBLAST* results to orient the paired-end reads. Poor quality bases with Qscore ≤3 were clipped using *NESONI Clip*^3^ that also removed reads with <25 bp remaining in either end. We then merged paired-end reads having ≥10 bp overlap with at least 90% identity, or having 9–19 bp overlap with at least 80% identity, using *SEQTK*. For heavy chain reads that failed, we raised the quality clipping to Qscore ≤10 and repeated the overlapping joining step. We separately processed the non-merged heavy chain reads after Qscore ≤10 clipping by concatenating the paired-end reads using *ILLUMINA PAIREDEND*.
(3) second *IGBLAST* and IMGT HighV-quest for joined reads: we ran a second in-house *IGBLAST* on the merged or concatenated reads to infer the germline V-gene and calculate the V-gene mutation frequency up to the highly conserved cysteine at the end of framework region (FR) 3. We retained the heavy chain reads with an alignment length of ≥275 bp, κ chain reads ≥245 bp, and λ chain reads ≥255 bp (including gaps) to ensure reliable alignment. Of the concatenated non-merged heavy chain reads, we only retained those aligned to a germline V-gene with a gap, reasoning that these reads failed the overlapping criteria due to the gap but not poor sequencing quality or mispairing. IMGT HighV-quest^4^ was then used to report CDR3 length and number of total and non-silent mutations in each FR and CDR1 and CDR2. From the IMGT HighV-quest results, we removed reads with stop codons, missing the cysteine at the end of FR3, or missing an IMGT-reported CDR3. To recover additional heavy chain reads with long CDR3s, we separately calculated the CDR3 length for all non-merged heavy chain reads after Q10 clipping, searching for the nucleotide motif 5′ GAYWBNGBBNYNTWYTWYTGY-*CDR3*-TGGGSYHVNGGVRYHNBVGTSDYYGTSKCY 3′ with the UNIX program *FUZZNUC*. Reads with a possible CDR3 length of 30 amino acids or more were then sent to IMGT HighV-quest to verify.
(4) plot results: from the second *IGBLAST* results, we plotted in GraphPad Prism 6 and R the data for V-gene family usage, individual V-gene usage, V-gene mutation frequency, number of FR and CDR mutations in V-gene, and CDR3 length, separately for μ, γ, α, κ, and λ chains. All above pipeline steps except for IMGT HighV-quest and Prism were command line-based, using R and shell scripts and the UNIX programs *IGBLAST, NESONI Clip, SEQTK, ILLUMINAPAIREDEND*, and *FUZZNUC*.
(5) Vetting. After the results from the pipeline were plotted, we selected reads with extreme values, or outliers, to manually vet. We extracted reads with the highest V-gene mutation values (reads within three percentage points of the maximum) and grouped them by V-gene family, aligning to a representative V-gene using Geneious v. 8.1. The reads were further clustered by CDR3 length, and clonal members with similar CDR3 sequences were retained. Singletons were checked within the processed data by searching for similar (>90% identity) CDR3 sequences. If reads were found with similar CDR3, indicative of clonal members, the outliers were retained; if not the singletons were removed. For reads with short (<3 amino acids) or long (>29 amino acids for heavy chains and >20 amino acids for light chains) CDR3 lengths, reads were extracted, aligned to a representative V-gene, and their CDR3 lengths were verified. Data plots were updated accordingly after vetting. For samples with multiple runs, data from each run were processed independently, and data plots were visualized for consistency before merging.

### Analysis of Previously Deposited 454 Pyrosequencing Data

To include additional HIV-1-infected individuals for SHM comparisons, we examined μ and γ chain reads from six previously published 454 pyrosequencing datasets (Table S2 in Supplementary Material) (27, 29, 33–35). Because these datasets were limited to the VH1 gene family, we used the VH1 data from our Illumina datasets for comparisons. To analyze the 454 reads, we removed those shorter than 300 bp and separated the μ and γ reads with a 24 bp motif unique to μ 5′ GGGAGTGCATCCGCCCCAACCCTT 3′ or γ 5′ GCCTCCACCAAGGGCCCATCGGTC 3′ constant regions, retaining reads with at least 21 bp identical to either motif. The reads were then processed using IGBLAST and IMGT HighV-quest. After outlier vetting as described above, the calculated VH1 SHM values were plotted. The common homopolymer errors in 454 pyrosequencing will not impact SHM calculations because our pipeline successfully aligns all reads to the germline V-genes with insertions and deletions that are excluded from the hypermutation calculation.

### Flow Cytometry and Single B Cell Sorting

Samples analyzed by NGS have also been phenotyped by flow cytometry. Briefly, blood donor PBMCs and cord blood cells were stained with two panels of antibody cocktails. The first panel included anti-human CD3-PE-CF594 (BD Biosciences, San Jose, CA, USA), CD19-PE-Cy7 (BioLegend, San Diego, CA, USA), CD20-APC-Cy7 (BioLegend), CD27-PerCP-Cy5.5 (BD Biosciences), IgG-FITC (BD Biosciences), IgM-V450 (BD Biosciences), IgD-PE (BD Biosciences), and IgA-APC (Miltenyi Biotec, San Diego, CA, USA); the second panel included anti-human CD3-APC-Cy7 (BD Biosciences), CD34-FITC (BD Biosciences), CD19-PE-Cy7, CD27-PE (BD Biosciences), CD38-APC (BD Biosciences), CD4-PE-CF594 (BD Biosciences), and CD8-PERCP (BD Biosciences). In addition, live/dead yellow stain (Invitrogen) was used to exclude dead cells. Fluorescence compensation was performed with anti-mouse Ig κ beads (BD Biosciences) stained with each antibody in a separate tube. After washing, cells were analyzed using a multi-laser flow cytometer LSR-II (BD Biosciences). Likewise, the HIS-CD4/B mice splenocytes were analyzed with markers as indicated using LSR-II, and the HIV-1+ PBMCs were analyzed using a multi-laser MoFlo sorter (Beckman Coulter, Jersey City, NJ, USA) contained with Biosafety Level 3 standards.

To sort individual B cells to recover unmutated IgM sequences, blood donor PBMCs and cord blood cells were stained with markers as indicated, and IgM+IgG−IgD+ B cells were singly sorted by the multi-laser MoFlo sorter into 96-well PCR plates containing 20 μl lysis buffer, consisting of 5 μl 5 × first-strand buffer (Invitrogen), 1.25 μl 0.1 M DTT, 0.5 μl RNaseOut, and 0.0625 μl Igepal (Sigma, St. Louis, MO, USA). The total content of cells passing through the sorter or LSR-II was analyzed with FlowJo (TreeStar, Cupertino, CA, USA). The PCR plates with sorted cells were frozen on dry ice and stored at −80°C.

From each sorted cell, the variable regions of IgM heavy and light chains were amplified by RT-PCR (36). Briefly, frozen plates with single B cell RNA were thawed at room temperature, and RT was carried out by adding into each well 3 μl random hexamers at 150 ng/μl (Gene Link, Hawthorne, NY, USA), 2 μl dNTP, each at 10 mM, and 1 μl SuperScript III (Invitrogen), followed by 42°C for 10 min, 25°C for 10 min, 50°C for 60 min, and 94°C for 5 min. After RT, 25 μl water was added to each well to dilute cDNA. The μ, κ, and λ chain variable regions were amplified independently by nested PCR in 50 μl, using 5 μl cDNA as template, HotStarTaq Plus DNA polymerase (Qiagen) and primers or primer mixes described (26, 36). Cycler parameters were 94°C for 5 min, 50 cycles of 94°C for 30 s, 52–55°C for 30 s, and 72°C for 1 min, followed by 72°C for 10 min. PCR amplicons were inspected on a precast 2% agarose gel (Embi Tec) and subjected to direct Sanger sequencing. Sequences were trimmed of low quality using Geneious v.8.1, followed by the in-house *IGBLAST* to include only unmutated sequences.

### NGS Analysis to Find Replicate Unmutated IgM B Cell Sequences

With the unmutated IgM sequences obtained by single-cell sorting and Sanger sequencing as described above, we searched for identical reads in MiSeq data from the same donor but different aliquots of cells. The final processed reads were used for this search by Geneious v.8.1 assembler, allowing at most two nucleotide differences for potential sequencing errors. For μ reads, we also searched against only the CDR3 sequence, allowing at most two nucleotide differences for sequencing error, to maximize the chance of finding identical VDJ recombination.

### Illumina MiSeq Data Deposit

The Illumina MiSeq sequence data used in this study have been deposited in the NCBI Sequence Read Archive^5^ under accession SRP111345.

### Statistical Analysis

R and GraphPad Prism 6 were used for statistical analysis. Two-tailed Wilcoxon rank sum tests were used to compare values between groups. Additionally, median distribution curves were calculated for V-gene mutation frequencies and CDR3 lengths of each study group and compared by Wilcoxon rank sum tests, using a mixed-effects model for each decile bin (e.g., Q0–Q10, Q10–Q20, Q20–Q30, etc.) of % total reads. The mixed-effects model was applied on the ranks of data with subject as a random effect and with group as a fixed effect, since each decile bin can contain multiple data measures from the same subject. *P* values of 0.05–0.1 were considered trends, and *P* values of <0.05 were considered statistically significant. FDR (false discovery rate) adjusted P values were also reported to correct for multiple testing.

## Results

### Basic Flow Cytometric Immunophenotyping

By flow cytometry, we analyzed the B cell phenotypes of 14 blood donor PBMCs, five HIV-1+ PBMCs, five cord blood, and three HIS-CD4/B mice. We first examined blood donor PBMCs for proportions of CD3−CD19+ B cells expressing IgM, IgD, IgG, or IgA, along with CD27 (Figures S1.1–S1.14 in Supplementary Material, panel 1). The majority (>60%) of B cells in blood donor PBMCs were IgM + or IgD+, followed by IgG+ and IgA+ B cells each at about 10%; CD27 expression was minimal on IgM+ or IgD+ cells, while about half of IgG+ and IgA+ cells expressed CD27, supporting CD27 as a memory marker (37). The precious HIV-1+ PBMCs were processed differently for antigen-specific B cells; included here are IgM, IgG, or CD27 staining data for CD3−CD19+ B cells (Figure S1.15 in Supplementary Material), which appeared phenotypically similar to those in blood donor PBMCs, with the caveat that a different flow cytometer was used. In cord blood cells (Figures S1.16–S1.20 in Supplementary Material, panel 1), however, IgG+ and IgA+ cells were sharply reduced to <1%, and accordingly, CD27 was hard to detect (38). For blood donor PBMCs and cord blood where excess cells were available, a second staining panel was included, examining human CD34 for stem cells that were absent in adult PBMCs but present in cord blood (Figures S1.1–S1.14 and S1.16–S1.20 in Supplementary Material, panel 2). In “panel 2” CD3−CD19+ B cells, we also examined CD27+CD38+ plasmablasts, which were generally lacking in both adult PBMCs and cord blood. In “panel 2” CD3+ T cells, the proportions of CD4+ T cells were generally greater than CD8+ T cells in both adult PBMCs and cord blood, with the exception of blood donor #5, of which the proportion of CD8+ T cells was greater than CD4+ T cells. The three HIS-CD4/B mice were analyzed using splenocytes collected at 26 weeks after human stem cell engraftment and all three mice had human CD3−CD19+ B cells (Figure S1.21 in Supplementary Material). While mouse #770 was naïve and lacked CD38+ plasmablasts, mouse #771 was sampled 9 days after injection with sheep red blood cells and mouse #755 was sampled 10 days after two immunizations of a malaria protein (22), thus both showed CD38+ cells, indicative of plasmablasts.

### Human BCR V-Gene Usage

**Figure 1:**
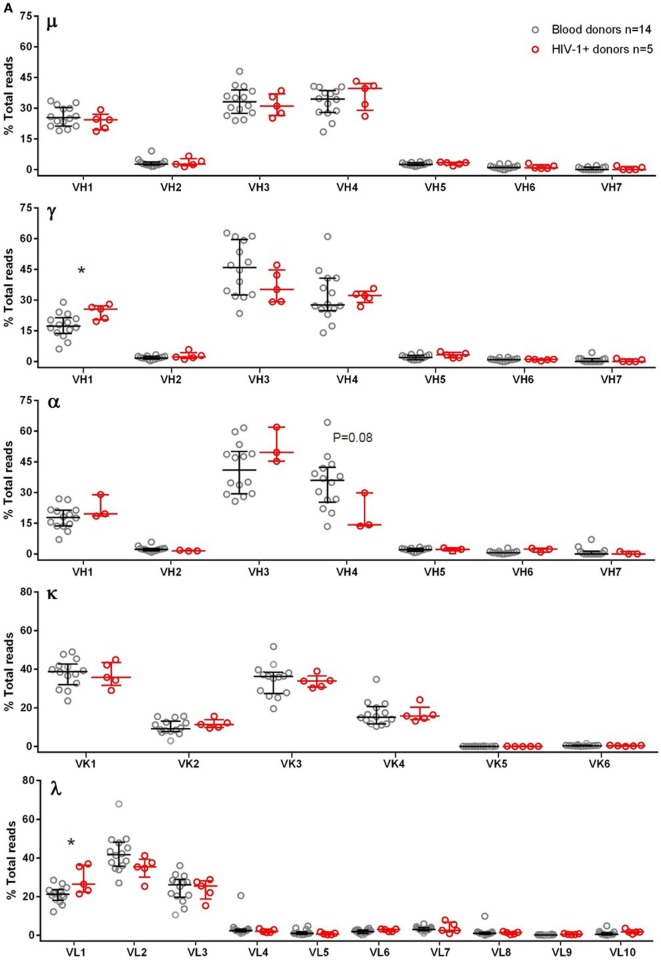

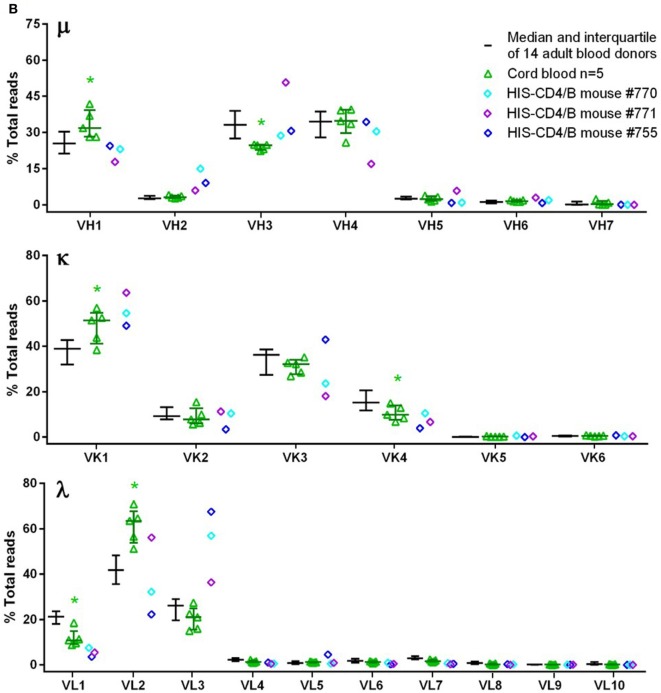
V-gene family usage for μ, γ, α, κ, and λ chains in blood donor PBMCs, HIV-1+ PBMCs, cord blood, and HIS-CD4/B mice. Each panel shows a heavy or light chain as indicated. The *y*-axis shows the frequency of sequence reads assigned to the V-gene families specified on the *x*-axis. **(A)** Comparisons between blood donors (gray circles) and HIV-1+ donors (red circles). Note that α data were only available for three HIV-1+ donors. **(B)** Comparison among adult blood donors (black bars), cord blood (green triangles), and three HIS-CD4/B mice (diamonds). Bars indicate the medians and interquartile ranges. Asterisks depict statistical significance between the indicated groups and the adult blood donors.

To maximize the use of cells, we amplified μ, γ, α, κ, and λ chains together, and the study samples were sequenced in a total of 22 MiSeq runs (Table 1), with some samples run multiple times. For samples with multiple runs, the data were consistent across runs and, therefore, combined for final plots. To characterize the authentic BCR repertoire, we intentionally applied the full data, including duplicate and clonally related sequences that are part of the BCR repertoire in the samples analyzed, particularly in clonally expanded IgG and IgA expressing cells. We first examined the V-gene family usage for μ, γ, α, κ, and λ chains among 14 blood donors (Figure 1A). For μ, γ, and α chains, the most commonly used were VH1, VH3, and VH4 families. For κ, the most commonly used belonged to VK1 and VK3, followed by VK2 and VK4. For λ, VL1, VL2, and VL3 were most used. We also examined five HIV-1+ subjects (Table S1 in Supplementary Material) for μ, γ, κ, and λ and found similar overall V-gene family usage between blood donors and HIV-1+ donors but with increased VH1 usage for γ and increased VL1 usage for λ (Figure 1A). We included α chain (or IgA) for only three HIV-1+ samples; based on these limited samples, the VH4 family showed a trend toward decreased usage compared to the blood donor controls (Figure 1A).

**Table 1:** Summary of MiSeq runs and numbers of sequences processed for each sample.

Subjects	Cell #^a^	B cell #^a^	Primers	Run #	Raw reads	Final reads	Subjects	Cell #^a^	B cell #^a^	Primers	Run #	Raw reads	Final reads
Blood donor #1	15 M	1.35 M	μ	4, 9, 11	7,234,822	3,957,846	AD344_58mpi	3 M	0.18 M	μ	10, 24	1,362,554	479,742
	10 M	1.0 M	γ	9, 11	666,546	290,189		3 M	0.18 M	γ	10, 24	3,374,395	1,251,819
	5 M	0.5 M	α	12	2,470,552	770,909		3 M	0.18 M	α	10, 24	1,571,286	533,054
	5 M	0.5 M	κ	9	1,951,904	1,345,002		3 M	0.18 M	κ	10, 24	2,450,664	1,395,331
	5 M	0.5 M	λ	9	1,778,239	1,153,322		3 M	0.18 M	λ	10, 24	3,499,457	1,536,322

Blood donor #2	10 M	0.5 M	μ	10, 16, 23	383,141	129,223	AD358_66mpi	4 M	0.02 M	μ	5	324,012	137,229
	10 M	0.5 M	γ	10, 16, 23	1,557,869	724,051		4 M	0.02 M	γ	5	605,277	317,465
	10 M	0.5 M	α	10, 16, 23	1,137,664	444,651		4 M	0.02 M	α	–	–	–
	10 M	0.5 M	κ	10, 16, 23	10,097,639	6,447,029		4 M	0.02 M	κ	5	4,091,836	3,503,178
	10 M	0.5 M	λ	10, 16, 23	5,771,862	2,588,383		4 M	0.02 M	λ	5	3,006,432	1,997,748

Blood donor #3	12 M	0.34 M	μ	3, 4, 9	3,987,630	2,478,027	MT1214	4 M	0.03 M	μ	10, 24	1,120,023	415,679
	7 M	0.2 M	γ	3, 9	2,582,656	1,129,031		4 M	0.03 M	γ	10, 24	4,613,380	1,550,904
	5 M	0.14 M	α	20	1,767,569	857,819		4 M	0.03 M	α	10, 24	2,110,698	596,901
	5 M	0.14 M	κ	3	5,232,876	3,911,691		4 M	0.03 M	κ	10, 24	2,535,838	1,401,831
	5 M	0.14 M	λ	3	3,687,638	2,034,512		4 M	0.03 M	λ	10, 24	4,344,878	2,108,511

Blood donor #4	10 M	0.43 M	μ	4, 13	2,086,363	1,218,647	MT6008	5 M	0.8 M	μ	5	237,425	95,535
	5 M	0.22 M	γ	13	762,532	278,578		5 M	0.8 M	γ	5	199,201	98,493
	5 M	0.22 M	α	13	1,323,831	549,050		–	–	α	–	–	–
	5 M	0.22 M	κ	13	1,790,492	1,268,685		5 M	0.8 M	κ	5	1,498,589	1,328,556
	5 M	0.22 M	λ	13	3,441,685	1,520,296		5 M	0.8 M	λ	5	1,173,145	784,482

Blood donor #5	10 M	0.47 M	μ	6, 11	5,481,458	2,376,246	MT8004	5 M	0.43 M	μ	12	702,468	222,171
	10 M	0.47 M	γ	6, 8, 11	5,926,836	1,173,265		5 M	0.43 M	γ	12	2,151,421	755,274
	5 M	0.24 M	α	12	2,035,405	622,928		5 M	0.43 M	α	12	1,142,749	394,573
	5 M	0.24 M	κ	6	1,687,774	1,141,919		5 M	0.43 M	κ	12	3,772,010	1,328,556
	5 M	0.24 M	λ	6	1,327,378	811,060		5 M	0.43 M	λ	12	4,558,654	1,288,007

Blood donor #6	10 M	0.25 M	μ	13, 15	2,301,445	627,933	HIV-1+ average *n* = 5			μ		749,296	270,071
	10 M	0.25 M	γ	13, 15	1,617,366	481,151				γ		2,188,735	794,791
	10 M	0.25 M	α	13, 15	3,202,540	1,369,673				α		1,608,244	381,132
	5 M	0.13 M	κ	13	4,656,261	1,660,315				κ		2,869,787	1,791,490
	5 M	0.13 M	λ	13	1,916,209	795,238				λ		3,316,513	1,543,014

Blood donor #7	5 M	0.16 M	μ	14	358,082	111,871	Cord blood #1	10 M	0.67 M	μ	10, 22	1,025,119	693,173
	5 M	0.16 M	γ	14	382,057	114,088		15 M	1.0 M	γ	10, 20, 22	2,555,492	24,743
	5 M	0.16 M	α	14	1,262,246	412,438		–	–	α	–	–	–
	5 M	0.16 M	κ	14	2,111,143	1,354,399		10 M	0.67 M	κ	10, 22	3,894,117	2,714,433
	5 M	0.16 M	λ	14	859,746	254,121		10 M	0.67 M	λ	10, 22	2,579,264	1,530,978

Blood donor #8	5 M	0.2 M	μ	16	1,093,257	211,774	Cord blood #2	4 M	0.31 M	μ	10, 22	3,464,112	1,767,568
	5 M	0.2 M	γ	16	645,074	51,831		4 M	0.31 M	γ	10, 22	2,339,633	4,180
	5 M	0.2 M	α	16	934,614	63,799		4 M	0.31 M	α	–	–	–
	5 M	0.2 M	κ	16	1,600,351	620,453		4 M	0.31 M	κ	10, 22	4,339,464	2,453,856
	5 M	0.2 M	λ	16	1,477,702	399,084		4 M	0.31 M	λ	10, 22	2,324,189	922,981

Blood donor #9	5 M	0.09 M	μ	17	173,619	92,793	Cord blood #3	5 M	0.31 M	μ	18	1,177,250	577,113
	5 M	0.09 M	γ	17	154,003	72,995		10 M	0.63 M	γ	18, 20	1,880,218	3,333
	5 M	0.09 M	α	17	180,656	69,599		–	–	α	–	–	–
	5 M	0.09 M	κ	17	3,693,606	2,797,240		5 M	0.31 M	κ	18	3,757,548	1,919,512
	5 M	0.09 M	λ	17	1,216,503	844,882		5 M	0.31 M	λ	18	2,326,314	1,007,509

Blood donor #10	5 M	0.24 M	μ	17	258,890	139,069	Cord blood #4	5 M	0.06 M	μ	21	1,997,207	1,068,831
	5 M	0.24 M	γ	17	314,471	149,968		6 M	0.08 M	γ	19, 21	4,184,127	21,495
	5 M	0.24 M	α	17	338,351	149,713		–	–	α	–	–	–
	5 M	0.24 M	κ	17	3,013,629	2,305,646		5 M	0.06 M	κ	21	1,527,369	1,028,046
	5 M	0.24 M	λ	17	1,017,566	701,235		5 M	0.06 M	λ	21	943,857	489,100

Blood donor #11	5 M	0.12 M	μ	21	260,228	132,777	Cord blood #5	5 M	0.23 M	μ	10, 23	1,366,763	648,805
	5 M	0.12 M	γ	21	560,996	184,635		5 M	0.23 M	γ	10, 23	639,519	3,321
	5 M	0.12 M	α	21	661,701	137,985		–	–	α	–	–	–
	5 M	0.12 M	κ	21	2,903,928	2,315,046		5 M	0.23 M	κ	10, 23	7,436,563	4,259,367
	5 M	0.12 M	λ	21	1,316,047	753,541		5 M	0.23 M	λ	10, 23	2,345,953	998,745

Blood donor #12	5 M	0.22 M	μ	21	311,518	144,219	Cord blood average *n* = 5			μ		1,806,090	951,098
	5 M	0.22 M	γ	21	489,286	215,173				γ		2,319,798	11,414
	5 M	0.22 M	α	21	543,340	230,793				α		–	–
	5 M	0.22 M	κ	21	2,569,322	2,024,760				κ		4,191,012	2,475,043
	5 M	0.22 M	λ	21	1,658,364	960,360				λ		2,103,915	989,863

Blood donor #13	5 M	0.23 M	μ	19	373,390	142,785	HIS-CD4/B mouse #770	4 M	0.15 M	μ	15	1,585,461	479,409
	5 M	0.23 M	γ	19	231,506	167,987		4 M	0.15 M	γ	15	1,076,565	331,611
	5 M	0.23 M	α	19	1,113,153	406,879		4 M	0.15 M	α	15	252,673	37,473
	5 M	0.23 M	κ	19	3,357,938	2,642,494		4 M	0.15 M	κ	15	2,361,858	1,526,127
	5 M	0.23 M	λ	19	2,276,048	1,509,054		4 M	0.15 M	λ	15	1,525,125	789,716

Blood donor #14	5 M	0.09 M	μ	25	556,342	512,021	HIS-CD4/B mouse #771	5 M	0.06 M	μ	15	292,732	89,443
	5 M	0.09 M	γ	25	818,692	297,710		5 M	0.06 M	γ	15	221,728	70,139
	5 M	0.09 M	α	25	1,560,758	711,215		5 M	0.06 M	α	15	149,731	12,892
	5 M	0.09 M	κ	25	2,583,508	2,381,360		5 M	0.06 M	κ	15	1,798,078	1,753,026
	5 M	0.09 M	λ	25	2,074,680	1,453,748		5 M	0.06 M	λ	15	1,501,346	881,750

Blood donor average *n* = 14			μ		1,771,632	876,002	HIS-CD4/B mouse #755	5 M	0.07 M	μ	14	513,814	146,630
			γ		1,175,208	380,759		5 M	0.07 M	γ	14	469,283	169,031
			α		1,311,278	485,532		5 M	0.07 M	α	14	77,609	19,453
			κ		3,261,773	2,301,146		5 M	0.07 M	κ	14	1,743,979	1,150,395
			λ		2,073,876	1,127,060		5 M	0.07 M	λ	14	1,321,204	824,756

*^a^Total cell and B cell numbers are reported in millions (M); B cell numbers were back calculated from the flow cytometry data of the corresponding samples*.

Because HIS-CD4/B mice contained reconstituted human CD4+ T cells and B cells, we sequenced the human BCR repertoires from the splenocytes of three HIS-CD4/B mice (Table 1). For comparison, we also sequenced the BCR repertoires from five human cord blood samples. Compared to adult PBMCs, the numbers of IgA and IgG expressing B cells in cord blood were sharply reduced as shown in Figures S1.16–S1.20 in Supplementary Material. Consequently, there was no specific α chain amplification by 5′ RACE PCR and only 10^3^–10^4^ γ chain sequences were recovered from each cord blood sample (Table 1). We thus only examined μ sequences for cord blood heavy chain V-gene usage. As in adult PBMCs, the most commonly used V-gene families in cord blood and HIS-CD4/B mice remained VH1, VH3, VH4, VK1, VK3, VL1, VL2, and VL3 (Figure 1B), with changes of increased VH1, VK1, and VL2 use and decreased VH3, VK4, and VL1 use in cord blood compared to adults (Figure 1B). Similar to cord blood, increased VK1 and decreased VL1 use was also noted in the three HIS-CD4/B mice, with additional increased VH2 and VL3 use relative to both cord blood and adults (Figure 1B).

**Figure 2:**
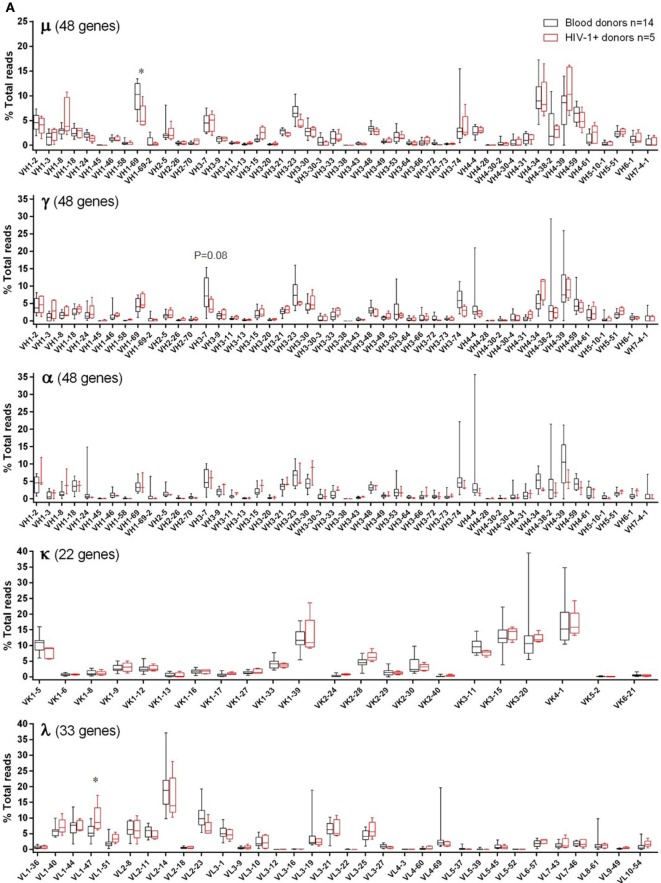

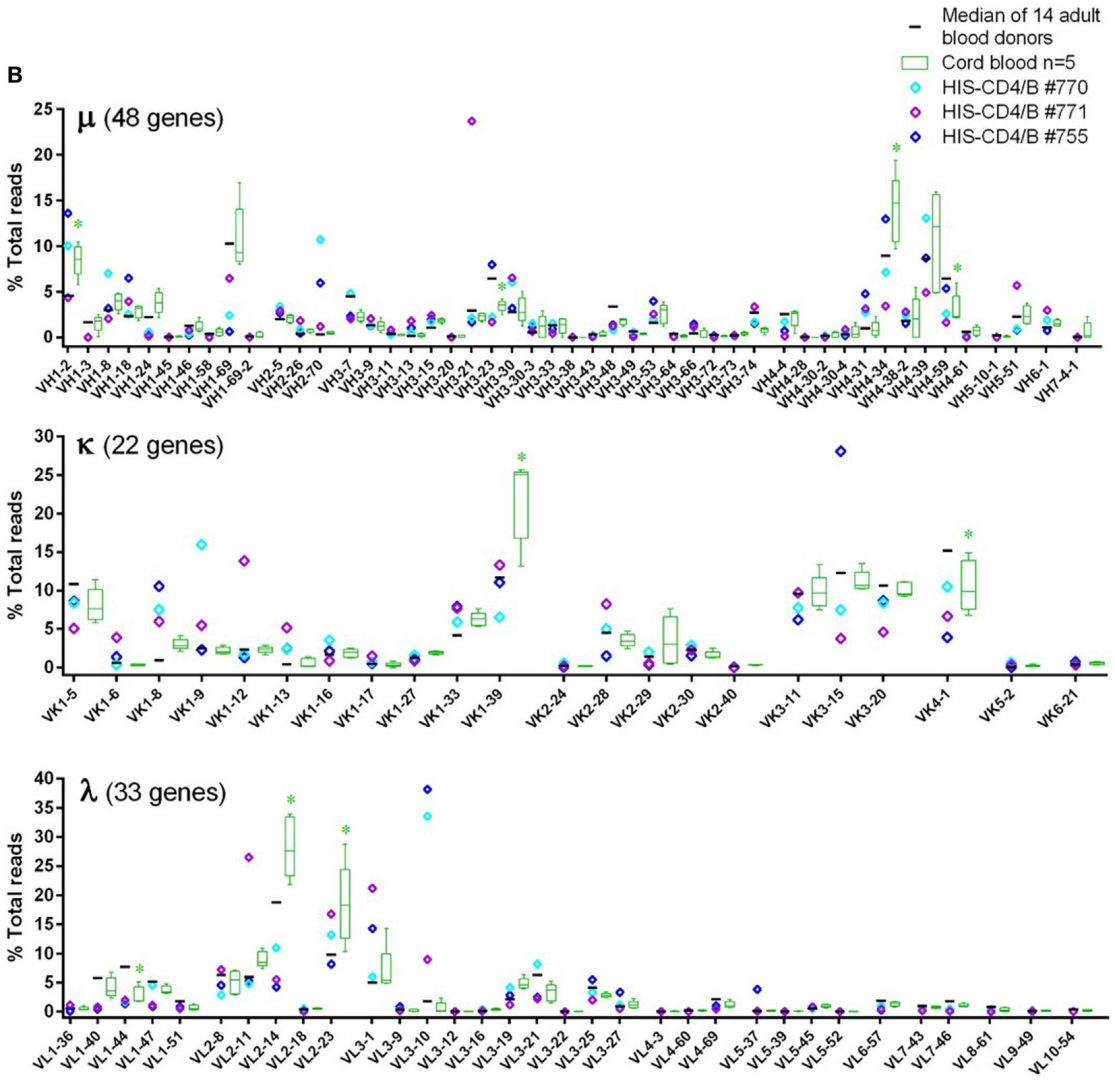
Individual V-gene usage of the μ, γ, α, κ, and λ chains in adult blood donor PBMCs, HIV-1+ PBMCs, cord blood, and HIS-CD4/B mice. Each panel shows a heavy or light chain as indicated. The *y*-axis shows the frequency of sequence reads assigned to the individual V-genes specified on the *x*-axis. **(A)** Comparisons between blood donors (gray) and HIV-1+ donors (red). Note that α data were only available for three HIV-1+ donors. The box plots indicate the medians, interquartile, and 10–90% ranges. **(B)** Comparisons among adult blood donors (black bars), cord blood (green boxes), and three HIS-CD4/B mice (diamonds). Asterisks depict statistical significant and >3% differences in the indicated genes between the indicated groups and the adult blood donors.

For individual V-gene usage, we only included functional human V-genes from the IMGT database (see text footnote 2). In adult blood donors, of 48 heavy chain V-genes, 8 were commonly used (≥5% in μ, γ, or α), namely VH1-2, VH1-69, VH3-7, VH3-23, VH3-74, VH4-34, VH4-39, and VH4-59 (Figure 2A). Of 22 κ V-genes, 6 were commonly used (≥10% each), including two VK1 genes (VK1-5 and VK1-39), all three VK3 (VK3-11, VK3-15, and VK3-20), and the only VK4 gene (VK4-1). Of 33 λ V-genes, VL2-14 was the most commonly used, followed by 9 other λ V-genes (>4% each), including VL1-40, VL1-44, VL1-47, VL2-8, VL2-11, VL2-23, VL3-1, VL3-21, and VL3-25. These V-genes were also commonly used in HIV-1+ samples, with overall usage similar to blood donor controls (Figure 2A). Statistical significant or trend differences of >3% were observed for VH1-69 in μ, VH3-7 in γ, and VL1-47 in λ. Though not significant, lower use of VH4-34 and VH4-39 in α (Figure 2A, middle α panel) might account for the trend of underused VH4 family in HIV-1+ samples (Figure 1A, middle α panel). The individual V-gene usage patterns of cord blood and HIS-CD4/B mice were generally similar to each other and consistent with those of adults (Figure 2B). However, compared to adults, increased use of VH1-2, VK1-39, VL2-14, and VL2-23 were observed in cord blood, with decreased use of VH3-23, VK4-1, and VL1-44, corresponding to changes in V-gene family use noted above. For HIS-CD4/B mice, we noted higher use of VH3-21, VK1-12, and VL2-11 in mouse #771, higher use of VK3-15 in mouse #755, and higher use of VL3-10 in both mice #770 and #755.

### Human BCR SHM

**Figure 3:**
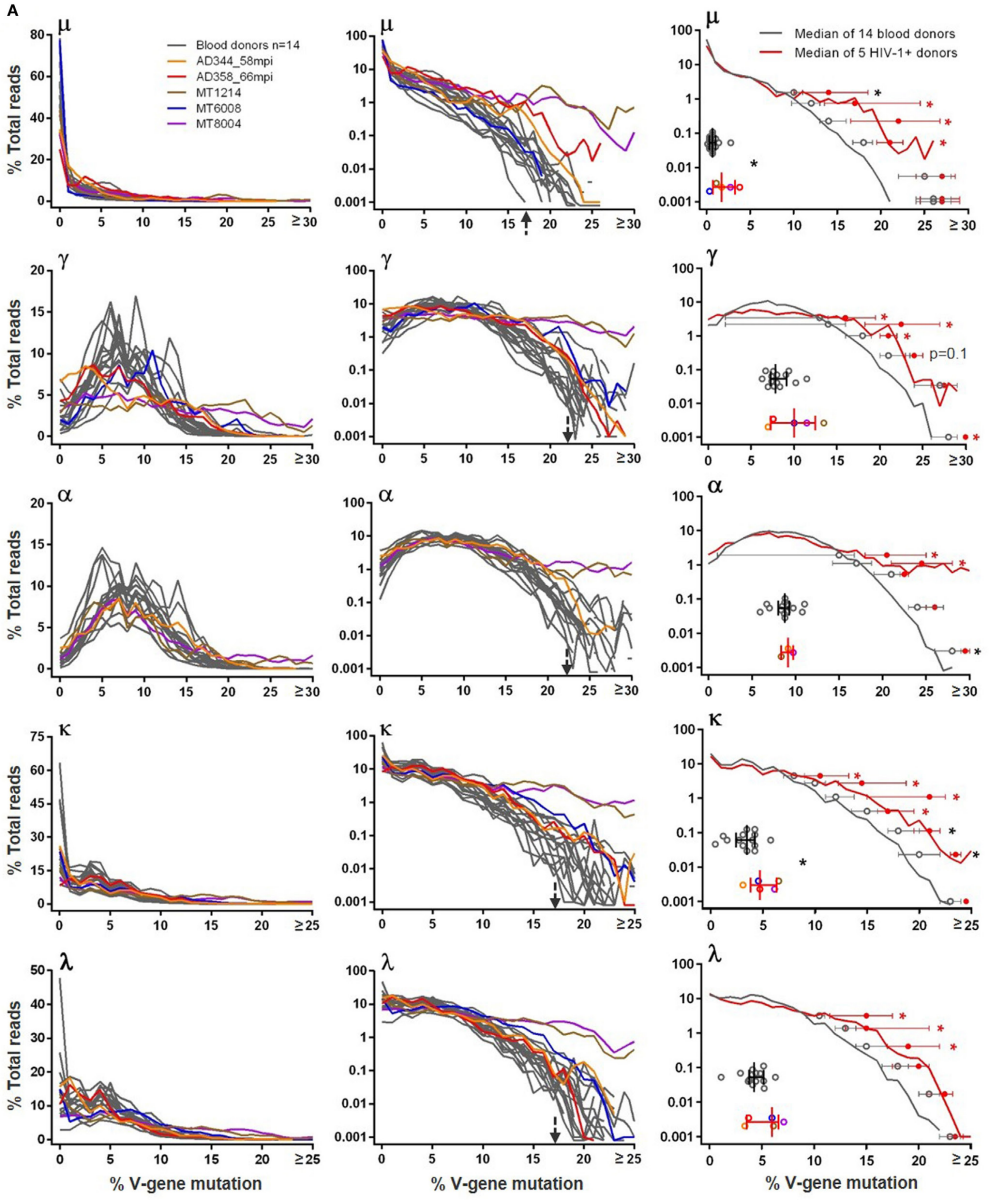

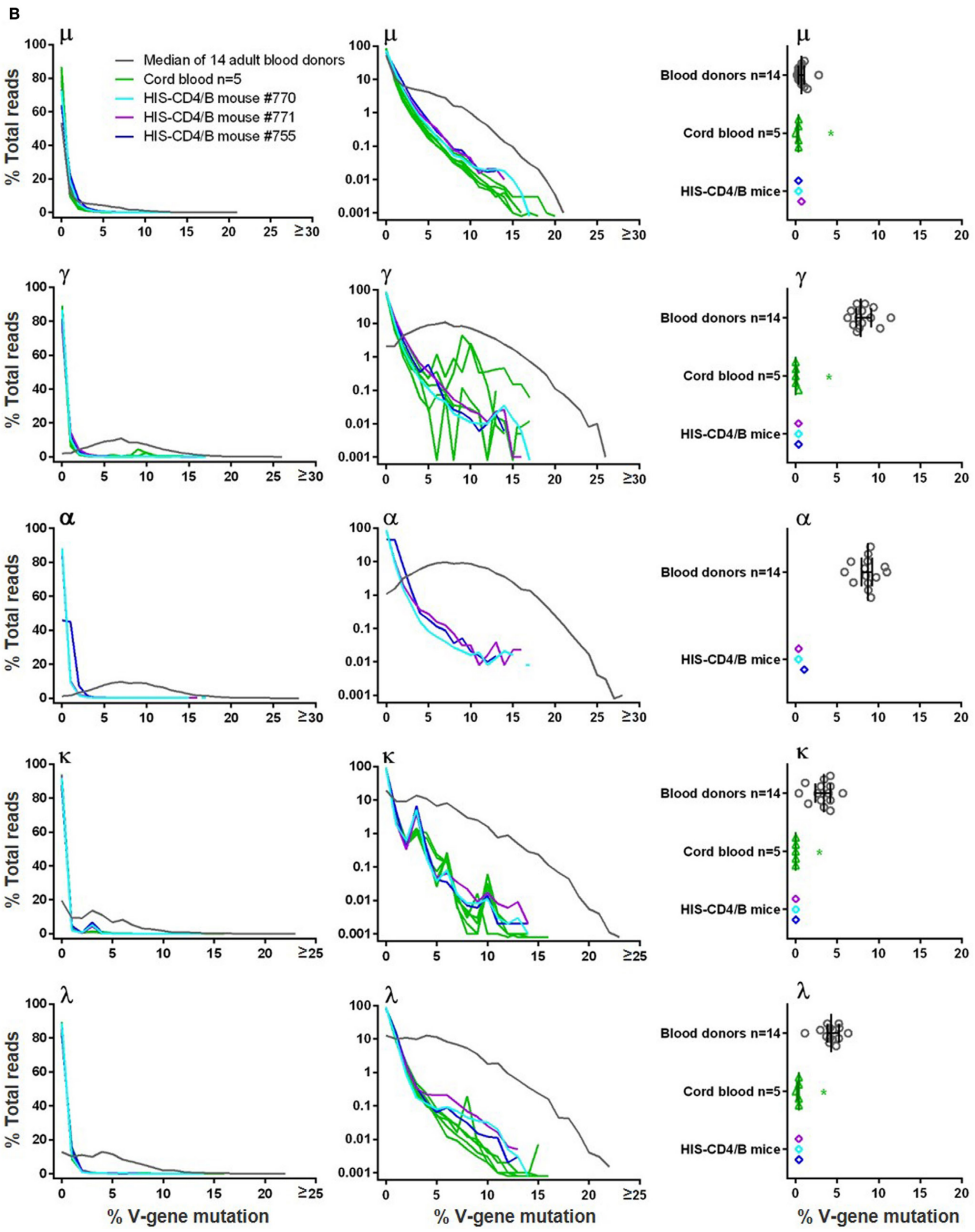
Heavy and light chain V-gene mutation frequencies in **(A)** blood donors versus HIV-1+ donors and in **(B)** adult blood donors versus cord blood and HIS-CD4/B mice. In left and middle panels, *y*-axis shows the % total reads (linear scale on left and log scale in middle) with indicated levels of mutations (in %) on *x*-axis. Gray lines indicate individual blood donors, and colored lines indicate individual HIV-1+ donors, cord blood samples, and HIS-CD4/B mice. Arrows indicate the mutation levels where the number of reads sharply dropped in blood donors. Right panels indicate comparisons of median mutation values (scatter plotted gray circles for blood donors and color circles for HIV-1+ donors, with median and interquartile ranges). Right panels in **(A)** also show median V-gene mutation frequency curves (gray for blood donors and red for HIV-1+ donors), with median and interquartile ranges indicated for each decile bin of % total reads where the two groups separate. Asterisks indicate significant differences, with red asterisks indicating significance based on FDR adjusted *P* values.

The BCR V-gene mutation frequency was calculated by aligning reads to their putative germlines. In blood donor PBMCs, we found that large portions (30–75%) of μ sequences were unmutated, likely originated from naïve B cells. The μ mutation frequency, similar across all 14 blood donors, did not follow normal distribution but followed a pattern with read numbers sharply tailing off with increased mutation frequencies (Figure 3A, top μ left panel). Roughly separated by a sudden drop of read numbers to <0.1% shown in the log plot (Figure 3A, top μ middle panel), hypermutated μ reads, >17% mutated, constituted a small fraction of total μ chains and were commonly found in blood donors, a finding not reported previously; in contrast, the previously reported μ chain mutation frequencies were 15% or less (37, 39, 40). For γ and α reads, the majority were hypermutated as expected, and their mutation frequencies in linear plots (Figure 3A, γ and α left panels) nearly followed the normal distribution, with peaks at 5–14%. Again, albeit at small fractions (<0.1%) shown in the log plots (Figure 3A, γ and α middle panels), hypermutated γ and α sequences contained >22% mutations, compared to >17% for μ chains, and were not well documented in healthy adults previously. Light chains were less mutated than heavy chains. In blood donor PBMCs, unmutated κ and λ reads were common, likely due to their origin from naïve B cells; mutated κ and λ reads showed peak mutation at 3–4% (Figure 3A, κ and λ left panels). Nonetheless, small fractions (<0.1%) of light chains were also heavily mutated, with >17% mutations in blood donor PBMCs shown in the log plots (Figure 3A, κ and λ middle panels).

Of the five HIV-1+ samples analyzed in this study, we plotted their μ, γ, α, κ, and λ V-gene mutation distributions on top of the 14 blood donor controls (Figure 3A). The mutation frequencies of MT1214 (in brown) and MT8004 (in purple) were the highest and apparently elevated compared to blood donor controls. To compare V-gene mutation frequency between blood donors and HIV-1+ donors, we first calculated the median mutation frequency for each individual and used Wilcoxon rank sum tests for comparison. The HIV-1+ samples showed higher median mutation frequencies than blood donors, reaching statistical significance for μ and κ chains but not for γ, α (with only three HIV-1+ samples) and λ chains (Figure 3A, right panels). To increase the sample size, we included μ and γ VH1 sequences from previously published 454 data (27, 29, 33–35) from six additional HIV-1 chronically infected individuals who developed bnAbs (Table S2 in Supplementary Material). Combined with the five HIV-1+ samples in this study, a total of 11 HIV-1+ samples showed higher VH1 μ and γ median mutation frequencies than blood donors, but the differences did not reach statistical significance (Figure S2 in Supplementary Material). We then plotted the median distribution curves for blood donors (in gray) and HIV-1+ samples (in red) and applied a mixed-effects model to compare mutation ranks for each decile bin of % total reads between the two groups (Figure 3A, right panels). The median mutation distribution curves for HIV-1+ samples were higher than those of blood donors at higher mutation frequencies across μ, γ, α, κ, and λ chains, with at least three decile bins showing significance; this was also the case for VH1 μ and γ comparisons with 11 HIV-1+ samples (Figure S2 in Supplementary Material). Together, these data supported increased V-gene mutation frequencies in HIV-1+ samples compared to blood donors.

The μ mutation frequencies of cord blood samples and HIS-CD4/B mice were similar to adults in that all μ sequences were primarily unmutated, although cord blood and HIS-CD4/B mice showed even lower μ mutation frequencies than those in adult PBMCs, with the cord blood data reaching statistical significance (Figure 3B, μ panels). However, unlike those in adult PBMCs, the vast majority of γ sequences were unmutated in cord blood (based on the limited number of γ sequences obtained from cord blood as specified in Table 1) and in HIS-CD4/B mice (Figure 3B, γ panels). For the three HIS-CD4/B mice with α data, the vast majority of α sequences were also unmutated in contrast to the hypermutated α sequences in adults (Figure 3B, α panels). Likewise, the three HIS-CD4/B mice and five cord blood samples showed greatly reduced light chain hypermutation relative to adults, with unmutated light chain sequences making up >90% of reads and few (~0.1%) having >4% mutations, as shown in the log plots (Figure 3B, κ and λ panels). The stark differences in median mutation frequencies all reached statistical significance for γ, α, κ, and λ between the primarily unmutated cord blood and the primarily hypermutated adult PBMCs (Figure 3B, right panels), obviating a need for the decile bin analysis.

For blood donor sequences, we separately examined the number of total and non-silent mutations in IMGT-defined FR1, CDR1, FR2, CDR2, and FR3 across V-gene (Figure S3 in Supplementary Material). For the primarily unmutated μ chain, mutation numbers were low across regions. For γ and α chains, mutations were concentrated in CDR1 and CDR2, followed by FR3 as the most mutated among FRs. For light chains, CDR1 remained more mutated than FRs; however, CDR2 mutations cannot be compared to other regions because the IMGT-defined light chain CDR2 typically contains only 9 bp—much shorter than the 30 bp assigned to CDR2. Among light chain FRs, FR2 appeared the most mutated. Non-silent mutations commonly made up 60–70% of total mutations, and the distribution patterns of the two agreed among regions. Comparing to blood donors, the HIV-1+ samples showed significant or trend increases in mutations in μ FR3, γ FR1, and κ all three FRs (Figure S3 in Supplementary Material). Hence, in agreement with a previous report (41), the elevated V-gene mutations in chronic HIV-1+ subjects with bnAbs were preferentially added in FRs, not in CDR1 and CDR2.

### Human BCR CDR3 Length

**Figure 4:**
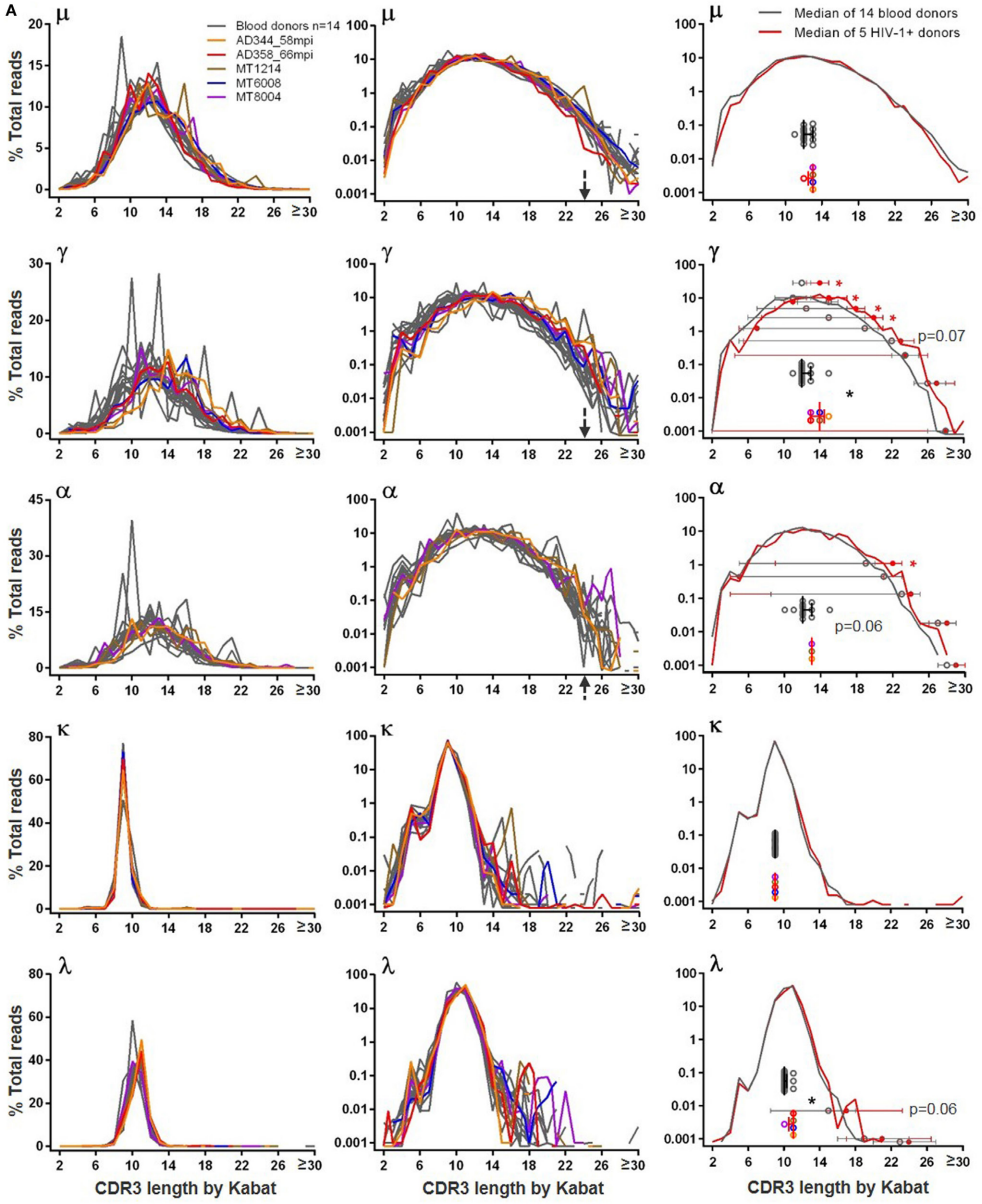

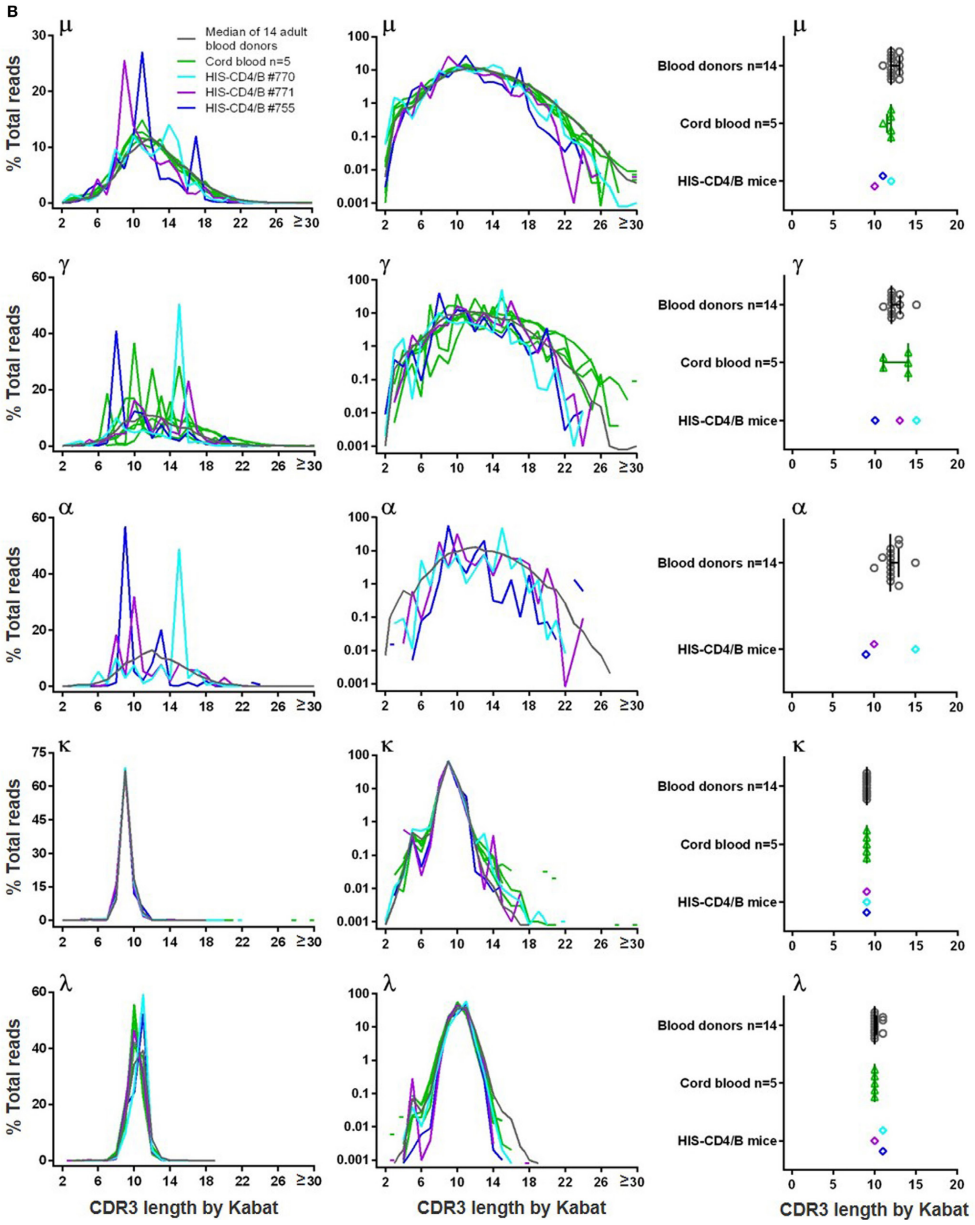
Heavy chain and light chain CDR3 length distribution in **(A)** blood donors versus HIV-1+ donors and in **(B)** adult blood donors versus cord blood and HIS-CD4/B mice. In left and middle panels, *y*-axis shows the % total reads (linear scale on left and log scale in middle) containing the CDR3 length indicated on *x*-axis. Gray lines indicate individual blood donors, and colored lines indicate individual HIV-1+ donors, cord blood samples, and HIS-CD4/B mice. Arrows indicate heavy chain CDR3 lengths where the number of reads sharply dropped in blood donors. Right panels indicate comparisons of median CDR3 lengths (scatter plotted gray circles for blood donors and color circles for HIV-1+ donors, with median and interquartile ranges). Right panels in **(A)** also show median CDR3 length distribution curves (gray for blood donors and red for HIV-1+ donors), with median and interquartile ranges indicated for each decile bin of % total reads where the two groups separate. Asterisks indicate significant differences, with red asterisks indicating significance based on FDR adjusted *P* values.

Because CDR3 is the most diversified region of BCR, we examined the CDR3 length distribution. In blood donors, the heavy chain CDR3 lengths were similar among μ, γ, and α sequences and followed the normal distribution (Figure 4A). The distributions were generally smooth, with few individuals having spikes, to a lesser extent for μ but more so for γ and α. All three classes of heavy chain CDR3 lengths peaked at 12 amino acids by Kabat definition. Long heavy chain CDR3s >24 amino acids were found in all analyzed blood donors at frequencies of <0.1% of total reads, and the longest reached 42 amino acids. For light chains, CDR3 lengths tightly clustered at 9 amino acids for κ and 10 or 11 amino acids for λ (Figure 4A). Light chain long CDR3s >20 amino acids were detected in <0.1% of total reads and were more common for κ than λ. Similar to blood donors, small numbers of sequences (<0.1%) in all examined HIV-1+ samples (Figure 4A) contained long CDR3s (>24 amino acids for heavy chains and >20 amino acids for light chains), and the longest heavy chain CDR3 reached 49 amino acids in one HIV-1+ sample. From the overlay plots, there was not an obvious difference between blood donors and HIV-1+ donors; however, compared to blood donors, the median CDR3 lengths were 1–2 amino acids longer for γ, α, and λ in HIV-1+ samples (Figure 4A, right panels). In agreement, the μ and κ median CDR3 length distribution curves overlapped between the two groups; the γ, α and λ median curves slightly shifted to the right (being slightly longer) for the HIV-1+ group, reaching statistical trend or significance for at least one decile bin of % total reads compared (Figure 4A, right panels).

In cord blood, because of the low numbers of IgG and IgA switched B cells, we focused on μ sequences, of which the CDR3 length distribution was highly similar to adults, peaking at 11–12 amino acids (Figure 4B). In contrast to the relatively smooth distributions of μ CDR3 length in adults and cord blood, the three HIS-CD4/B mice each showed one to two spikes at certain CDR3 lengths, suggesting a potential selection of B cells with these CDR3 lengths in these mice (Figure 4B). This difference was more obvious for γ and α chains. Based on the limited numbers of γ sequences from cord blood, 4 out of 5 cord blood samples each showed one to two spikes at certain CDR3 lengths; likewise, each HIS-CD4/B mouse also showed at least one spike for γ and α (Figure 4B). Therefore, compared to adults, class-switched B cell clones in cord blood and HIS-CD4/B mice appeared relatively less diversified based on their less continuous CDR3 lengths. Despite these differences, HIS-CD4/B mice and cord blood did contain small numbers (<0.1%) of heavy chain CDR3s reaching 24 amino acids and longer, a property shared with the adult BCR repertoire. Light chain CDR3 length distributions in HIS-CD4/B mice and cord blood were similar to those of adults, with rare long light chain CDR3s >20 amino acids found for κ but not for λ (Figure 4B, κ and λ panels).

### Searching for Unmutated IgM BCR Replicates in NGS

**Figure 5:**
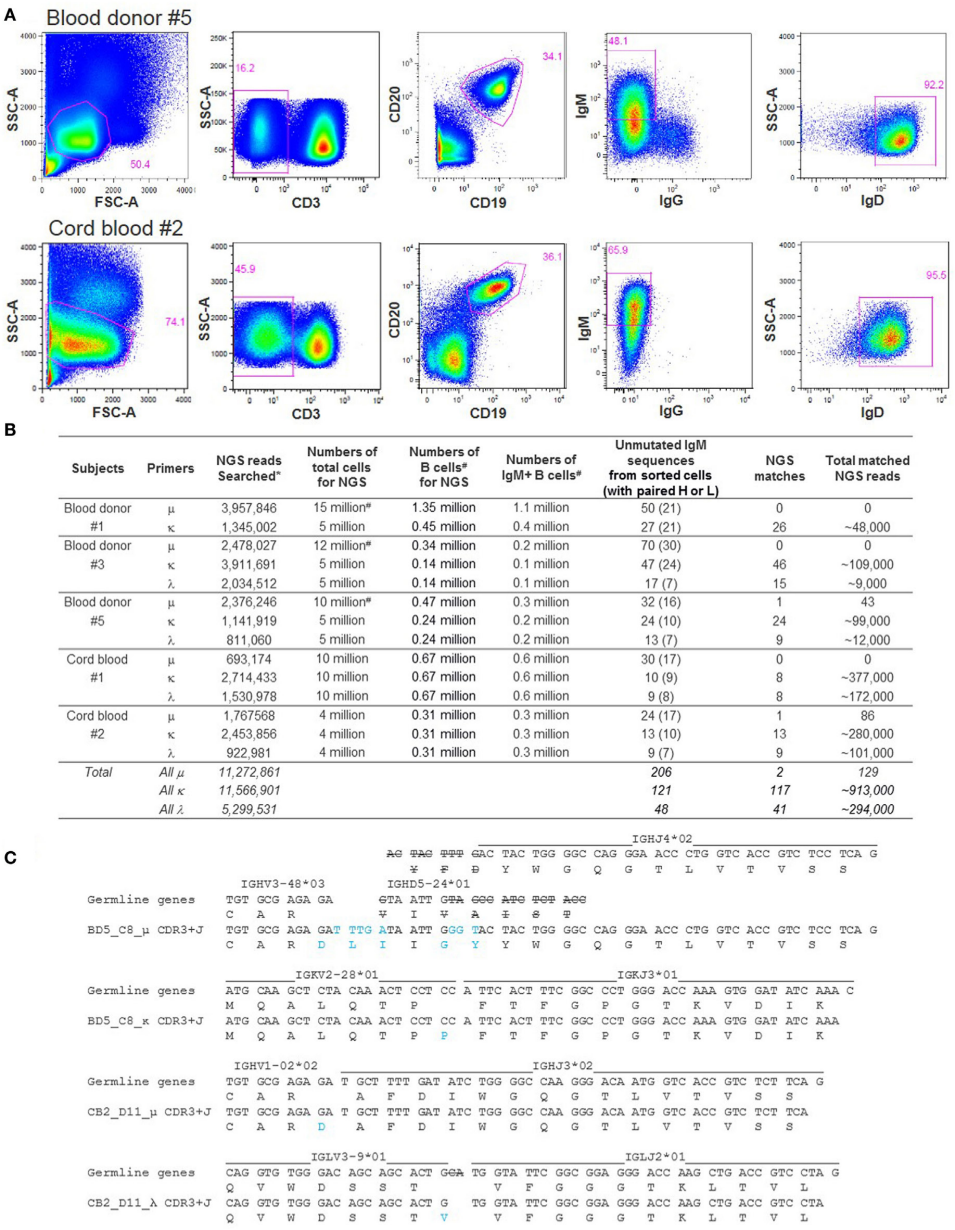
Next-generation sequencing (NGS) searches for unmutated IgM sequences obtained by single-cell sorting. **(A)** Representative FACS of individual IgM+IgG−IgD+ B cells from blood donor #5 PBMC and from cord blood #2. About 160 cells passing through gate CD3−CD19+IgM+IgG−IgD+ were sorted from each sample. SSC-A, side scatter area; FSC-A, forward scatter area. **(B)** Summary of the NGS searches for unmutated IgM sequences obtained by single-cell sorting. Symbol * notes that the NGS searches used the final processed reads and thus match the number of final reads in Table 1. Symbol ^#^ notes that the μ reads were combined from two to three sequencing runs and that the B cell and IgM+ cell numbers were back calculated from the flow cytometric data of the corresponding samples. **(C)** Analysis of V(D)J rearrangement and junction of the two unmutated IgM sequences identified both by single B cell sorting and NGS. Strikeouts indicate tentative exonuclease activity, and blue font indicates tentative junction sequences.

To examine the possibility of identifying unmutated IgM BCR replicates in NGS, we first sorted IgM+IgG−IgD+ B cells (Figure 5A) from blood donors #1, #3, and #5, and cord blood samples #1 and #2, and recovered dozens of unmutated IgM BCR sequences from each sample, with paired heavy and light chains (Figure 5B). These random B cell sortings did not yield any identical or replicate unmutated μ chains but occasionally recovered a few identical κ or λ light chain sequences. We then searched 24–70 unique unmutated μ sequences, 10–47 κ, and 9–17 λ sequences from each subject for replicate reads in the NGS data generated from a separate aliquot of cells. The numbers of cells used to generate NGS reads were estimated to be 0.1–1.1 million IgM+ B cells (Figure 5B). We successfully found replicates in NGS for most of the light chain sequences searched: 117 out of 121 unmutated κ and 41 out of 48 unmutated λ (Figure 5B). The number of replicate NGS reads for each light chain was commonly large, from hundreds to thousands. Searches for matching μ sequences, however, were much more challenging. Among the five samples examined, we found matching NGS reads from two samples, blood donor #5 and cord blood #2, each with a single unmutated μ sequence, BD5_C8_μ and CB2_D11_μ, respectively. The number of replicate μ reads was 43 matching BD5_C8_μ and 86 matching CB2_D11_μ (Figure 5B). As expected, we also found in NGS identical light chain sequences paired with these two heavy chains. The number of replicate reads was 44 matching BD5_C8_κ and >1,000 matching CB2_D11_λ. Of the two B cells, we analyzed the V(D)J rearrangements of the paired μ and light chain sequences, verifying that both were unmutated BCRs (Figure 5C). Interestingly, different from the classical heavy chain VDJ rearrangements, junction analysis of CB2_D11_μ suggested a possible VJ rearrangement without D. To our knowledge, this type of sequence configuration has never been reported for BCR heavy chains. Heavy chains rearranged from direct V to J joining have only been reported in a D-gene-less mouse model (42). Our finding of a physiological example supports the possibility of this unusual sequence configuration and calls for further investigation. We note that the CB2_D11_μ sequence came from a cord blood sample; whether this type of heavy chain is unique to cord blood remains to be determined. Given the observation that the NGS data contained replicates for many light chains but not their paired μ chains, these data suggest that it is common for unmutated IgM+ cells to share identical light chains.

## Discussion

Advances in unbiased PCR and NGS technologies have made the characterization of the immense BCR repertoire more realistic. Here we used 5′ RACE PCR and Illumina MiSeq 2 × 300 bp paired-end indexed sequencing to characterize the human reference BCR from 14 blood donor PBMCs, reporting the BCR features of V-gene usage, V-gene mutation frequency, and CDR3 length using reads spanning the full variable region, with parallel immunophenotyping. In addition to γ chain, we included the less studied μ, α, κ, and λ chains and found that the μ, κ, and λ chains each exhibited specific characteristics, and that the α chain characteristics were broadly similar to γ. Because of individual variations, the exact ranking of the use of each V-gene family varied among individuals. For example, though VH3 was the most commonly used in most individuals, VH4 could be more commonly used in some individuals. The individual variations emphasized the need of sampling multiple individuals to establish a reference BCR repertoire. Because genomic DNA was not sequenced, we were unable to associate the V-gene usage to gene duplications or deletions, which have been identified as common in human immunoglobulin heavy chain locus (43). We were also limited in that the age and sex of the studied blood donors was unknown. For V-gene mutation frequencies, the large number of reads by Illumina MiSeq allowed for examining the extremes that have been missed or neglected in healthy adults. For SHM distributions across V-gene regions (by IMGT definition), blood donor sequences exhibited higher mutation frequencies in CDRs than FRs, as demonstrated long ago (44, 45). As high levels of antibody SHM have been viewed as an unusual feature of HIV-1 bnAbs (46, 47), our findings of highly mutated BCRs in blood donors suggest that common antigens that all humans encounter, or persistent viruses that all humans harbor, may also elicit B cell responses producing highly mutated antibodies or B cell memory. Thus, high levels of SHM seen in chronic HIV-1 and HCV infections (48, 49) are not so “unusual” or “atypical.” The CDR3 lengths were similar across μ, γ, and α chains, suggesting that class switching from IgM to IgG and IgA does not directly involve modifications in CDR3 length, and that individual IgG and IgA clonal expansions do not change the overall CDR3 length distribution. Long CDR3s were commonly found with >24 amino acids for heavy chain and >20 amino acids for light chain, particularly in κ sequences. These long κ CDR3s were discontinuous in distribution, and many appeared to be duplication of two CDR3s joined together. While multiple mechanisms including duplication of D segments (50–52) have been reported for long heavy chain CDR3 formation, mechanisms for long light chain CDR3s have not been addressed.

With the BCR reference established from 14 blood donors, we next compared it with five HIV-1+ donors. Though plasmablasts and B cell memory subsets have been reported altered in some HIV-1+ subjects (53, 54), basic immunophenotyping of the general B cell compartment did not show a difference between HIV-1+ donors and blood donors. HIV-1 infection generally did not change V-gene use, with exceptions of lower VH1-69 use in μ, lower VH3-7 use in γ, and higher VL1-47 use in λ. Because VH1-69 has been shown to frequently encode high titers of HIV-1 antibodies directed to the CD4-induced co-receptor-binding site (55–57), we speculate that the lower proportions of VH1-69 μ chains in HIV-1+ samples may reflect a preferential activation of VH1-69-encoding naïve B cells followed by a subsequent class switching to IgG, leading to an exhaustion of VH1-69-encoding naïve B cells in the repertoire. The mechanisms behind decreased VH3-7 γ and increased VL1-47 λ in HIV-1+ samples are unclear, though it has been shown that VH3-7 encodes an IgG1-switched HIV-1 bnAb VRC-PG05 that recognizes the immunological silent face of HIV-1 gp120 (58), and VL1-47 encodes a HIV-1 bnAb 1B2530 that likely targets the CD4 receptor-binding site (26). While HIV-1 infection might not change the levels of SHM immediately, chronically infected individuals showed elevated mutation frequencies compared to blood donor controls, and the elevated V-gene mutations were preferentially added in FRs, not in CDR1 and CDR2, in line with a study showing FR mutations are required for HIV-1 bnAbs (41). The elevated SHM of IgG in HIV-1 chronic infection may occur with insertions and deletions (59, 60); if in CDR3, insertions and deletions will change the CDR3 lengths. Indeed, we found slightly longer median γ, α, and λ CDR3s in HIV-1+ donors relative to blood donors. These results are concordant with the importance of long heavy chain CDR3 in some HIV-1 bnAbs, allowing for reaching to deep and partially obscured regions of the HIV-1 envelope trimer (61, 62).

For near-term fetuses and newborns, previous studies (63–68) reported similar V-gene usage and CDR3 length to adults but rare class-switching and lower SHM levels. Our study is the first using 5′ RACE and Illumina MiSeq for cord blood analysis with parallel immunophenotyping. Cord blood in a large part recapitulated the V-gene usage in adults. HIS-CD4/B mice also shared the same commonly used V-genes, indicating successful reconstitution of the overall human BCR repertoire in this mouse model. However, the V-gene mutation frequencies from cord blood or HIS-CD4/B mice were sharply reduced compared to adults. Though the impact of these differences for B cell function and antibody response is uncertain (69), the overall “naïve” BCR repertoires in humanized mice have not been recognized previously because of absence of data. Unlike those in adults, the heavy chain CDR3 length distributions in cord blood and especially in HIS-CD4/B mice were less smooth and less continuous, suggesting a potential over representation of recombined BCRs with certain CDR3 lengths. While HIS-CD4/B mice (22) may serve as a useful model for human immunological studies, these differences and limitations need to be taken into consideration.

With the available NGS data, we commonly found replicates for unmutated light chain sequences, likely due to the relatively limited diversity in light chains with only VJ recombination and some naïve B cells sharing common light chains; however, it was extremely difficult to find replicate unmutated μ chain sequences, likely due to the high sequence diversity of heavy chains resulting from VDJ recombination with unique D-genes and V-D/D-J junctions, and also due to the limited absolute numbers of IgM+ B cells (0.1–1.1 million) used to generate the NGS data. Albeit rare for μ chains and limited cells sampled, we found two unmutated μ chains and their paired light chain sequences in the NGS data with replicate reads. If these unmutated μ chains belong to naïve B cells, this finding would provide the first sequence evidence supporting that naïve B cells in peripheral blood are present as clones of cells, not as unique cells without clonal members. Though unlikely, we cannot rule out the possibility of independent identical VDJ rearrangements, given that a small number of identical BCR or heavy chain CDR3 sequences have been reported from different individuals (9, 70, 71). Nonetheless, B cells bearing identical BCRs will likely function similarly regardless of homeostatic expansion or independent identical VDJ recombination. A recent study using a germline-targeting immunogen (72) sampled over 60 million naïve B cells from 15 healthy donor PBMCs to recover 27 naïve BCR sequences of interest, and none was reported to be identical to each other, although the study did not generate NGS data. To fully demonstrate the presence of identical naïve BCRs in peripheral blood, a more focused study sampling a larger number of B cells would be required. Our initial finding of two clonal unmutated IgM+ B cells implies that, to track B cell lineages of interest back in time, such as tracking HIV-1 bnAb lineages (73–75), it is possible, though extremely challenging, to identify by NGS the corresponding unmutated IgM sequences, especially with limited amount of specimens.

While advances in NGS provide deeper insight into the BCR repertoire, limitations still exist. The Illumina 2 × 300 bp paired-end sequencing, when combined with 5′ RACE, still does not provide full-length coverage of all BCR variable regions, particularly for heavy chains with long CDR3. For example, using our bioinformatics workflow, while >70% of paired-end light chain reads were overlapping (indicating full-length coverage), only about 50% of paired-end heavy chain reads were overlapping. To address this problem, our workflow included non-overlapping heavy chain reads to specifically recover those with long CDR3s. Our findings of long CDR3 reads demonstrate the efficacy of our approach despite the sequencing length limitation. Another caveat to the sequencing method here is the loss of paired heavy and light chains, and thus naturally expressed antibodies or BCRs cannot be reconstituted based on the NGS data alone. Though not yet readily available, new sequencing platforms are being developed to address this issue (76, 77). As of today, NGS combined with single B cell sorting and sequencing, which maintains the heavy and light chain pairing information, is still the most practical and comprehensive system to study B cell lineages of interest. Furthermore, depending on the specific scientific questions, we did not separate identical or clonally related reads from unique reads that define individual B cell clones, and bioinformatics algorithms specific for this purpose have been developed (12, 78). Finally, we did not incorporate unique molecular identifiers at the RT step, which may help to address PCR and sequencing errors (79).

In summary, we established the reference BCR repertoire for μ, γ, α, κ, and λ chains in healthy adult PBMCs and cord blood cells, with basic B cell flow cytometry, providing B cell phenotype and BCR sequence information to be compared with those of diseases and humanized animal models for potential changes. Importantly, our results revealed sequence features that have been vaguely reported or underappreciated. The BCR repertoire comparison, however, is different from tracking B cell lineages over time. Since longitudinal samples are not always available, healthy controls or reference BCRs still set the standard to inform potential alterations in diseases at the repertoire level.

## Ethics Statement

Adult blood and cord blood samples were purchased from the New York Blood Center. The HIV-1-infected PBMC samples were obtained with Institutional Review Board (IRB) approved protocols. The HIS-CD4/B mice study protocol was approved by the Institutional Animal Care and Use Committee (IACUC) at The Rockefeller University (Assurance #A3081-01).

## Author Contributions

XW and EW designed the study. EW prepared samples for Illumina sequencing and carried out computational bioinformatics analyses. MJ performed flow cytometry analysis and single-cell sorting of human cells. CJ performed statistical analysis. HL isolated adult PBMCs and cord blood cells. AF, DK, and MM provided HIV-1+ human PBMCs. JH and MT developed the HIS-CD4/B mouse and provided mouse splenocytes, of which the flow cytometry analysis was performed by CF. XW and EW wrote the paper, on which all authors commented.

## Conflict of Interest Statement

The authors declare that the research was conducted in the absence of any commercial or financial relationships that could be construed as a potential conflict of interest.

## Footnotes

^1^http://www.ncbi.nlm.nih.gov/projects/igblast/.

^2^http://www.imgt.org/.

^3^http://www.vicbioinformatics.com.

^4^http://www.imgt.org/HighV-QUEST.

^5^https://www.ncbi.nlm.nih.gov/sra.
