# Supplementary material for: 5′ Rapid Amplification of cDNA Ends and Illumina MiSeq Reveals B Cell Receptor Features in Healthy Adults, Adults With Chronic HIV-1 Infection, Cord Blood, and Humanized Mice
Source: Front Immunol. 2018 Mar 26;9:628. doi: 10.3389/fimmu.2018.00628 (PMC5879793; doi:10.3389/fimmu.2018.00628)
Supplement: Supplementary file 1 [file Presentation_1.PDF]

Supplementary Material

5' Rapid Amplification of cDNA Ends and Illumina MiSeq Reveals B Cell Receptor Features in Healthy Adults, Adults with Chronic HIV-1 Infection, Cord blood, and Humanized Mice

Eric Waltari[1], Manxue Jia[1], Caroline S. Jiang[2], Hong Lu[1], Jing Huang[1], Cristina Fernandez[1], Andrés Finzi[3], Daniel E. Kaufmann[3,4], Martin Markowitz[1], Moriya Tsuji[1], Xueling Wu[1*]

[1]Aaron Diamond AIDS Research Center, Affiliate of The Rockefeller University, New York, New York, USA.

[2]Hospital Biostatistics, The Rockefeller University, New York, New York, USA.

[3]Centre de Recherche du CHUM and Université de Montréal, Montreal, QC, Canada.

[4]Center for HIV/AIDS Vaccine Immunology and Immunogen Discovery (CHAVI-ID), La Jolla, CA, USA.

*** Correspondence:** Corresponding Author: xwu@adarc.org

Supplementary Figures

Supplementary Figure 1.1-21 Basic flow cytometric phenotyping of 14 blood donor PBMCs, 5 HIV-1+ PBMCs, 5 cord blood cells, and 3 HIS-CD4/B mice by splenocytes.

Supplementary Figure 2. Comparison of VH1 mutation frequencies in 14 blood donor controls versus 11 HIV-1 infected individuals, including previously published 454 data from 6 HIV-1+ individuals.

Supplementary Figure 3. Violin plot of mutation numbers by IMGT-defined V-gene regions for μ, γ, α, κ and λ.

Supplementary Tables

Supplementary Table 1. Summary of the HIV-1+ subjects.

Subject ID	Cohort, HIV-1 subtype	Infection time	Plasma viral load (copies/ml)	CD4 Count (cells/µl)	Plasma neutralization
AD344_58mpi	ADARC, B	58 months	35,700	533	broad
AD358_66mpi	ADARC, B	66 months	8,520	734	broad
MT1214	Montreal, B	21 years	17,902	186	broad
MT6008	Montreal, B	12 years	50	800	broad
MT8004	Montreal, B	9 years	87	970	broad

Supplementary Table 2. Summary of published 454 data sets included in analyses.

Subject ID	HIV-1 bnAb ID	Primers	SRA archive numbers	Reference	Final reads
IAVI74	VRC-PG04	VH1 μ, γ	SRR275711, SRR277211	27	μ=175,275<br>γ=431,617
NIH45_1995	VRC01	VH1 μ, γ	SRR1767446	29	μ=51,663<br>γ=265,666
C38	VRC18	VH1 μ, γ	SRR924015, SRR924016<br>SRR924017	33	μ=129,261<br>γ=552,561
NIH44	VRC13	VH1 μ, γ	SRR1818726	34	μ=138,877<br>γ=163,593
RU01	8ANC131	VH1 μ, γ	SRR1818729	34	μ=89,397<br>γ=188,291
CH0219	VRC-CH31	VH1 μ, γ	SRR2819798	35	μ=41,076<br>γ=71,960

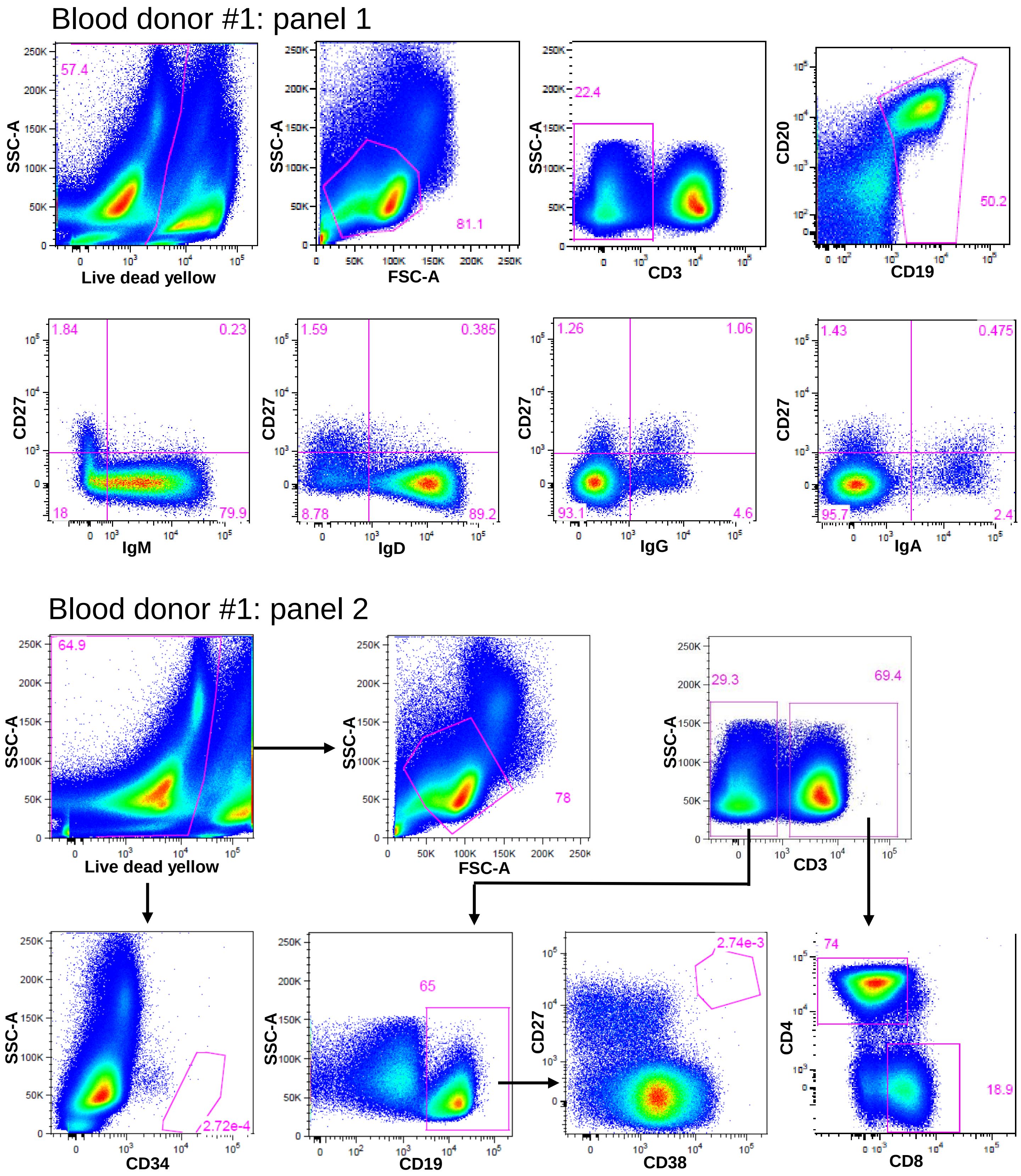

Supplementary Figure 1.1 Basic flow cytometric phenotyping of PBMCs from blood donor #1. In "panel 1", cells in CD27 plots are from the CD3-CD19+ gate. SSC-A, side scatter area; FSC-A, forward scatter area.

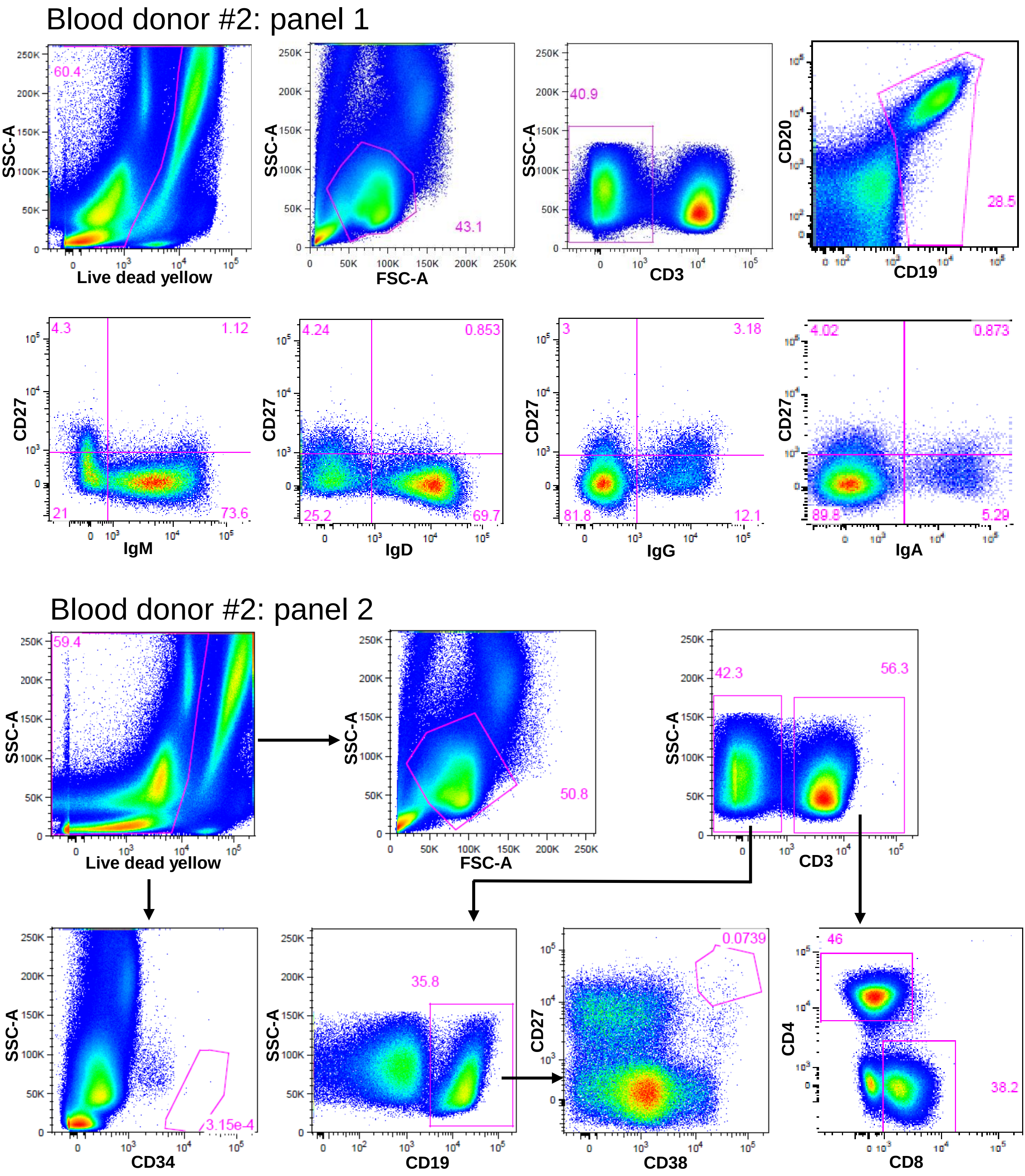

Supplementary Figure 1.2 Basic flow cytometric phenotyping of PBMCs from blood donor #2. In “panel 1”, cells in CD27 plots are from the CD3-CD19+ gate. SSC-A, side scatter area; FSC-A, forward scatter area.

Blood donor #3: panel 1

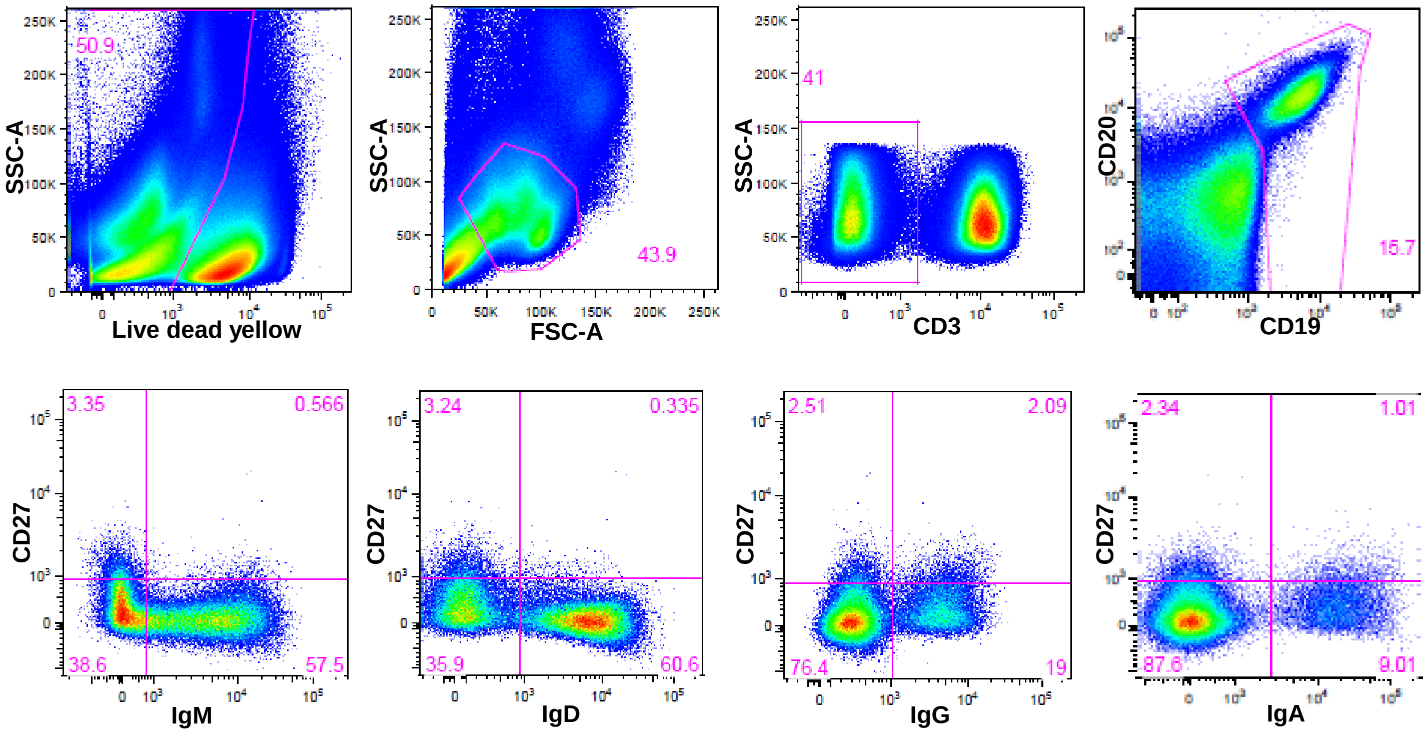

Blood donor #3: panel 2

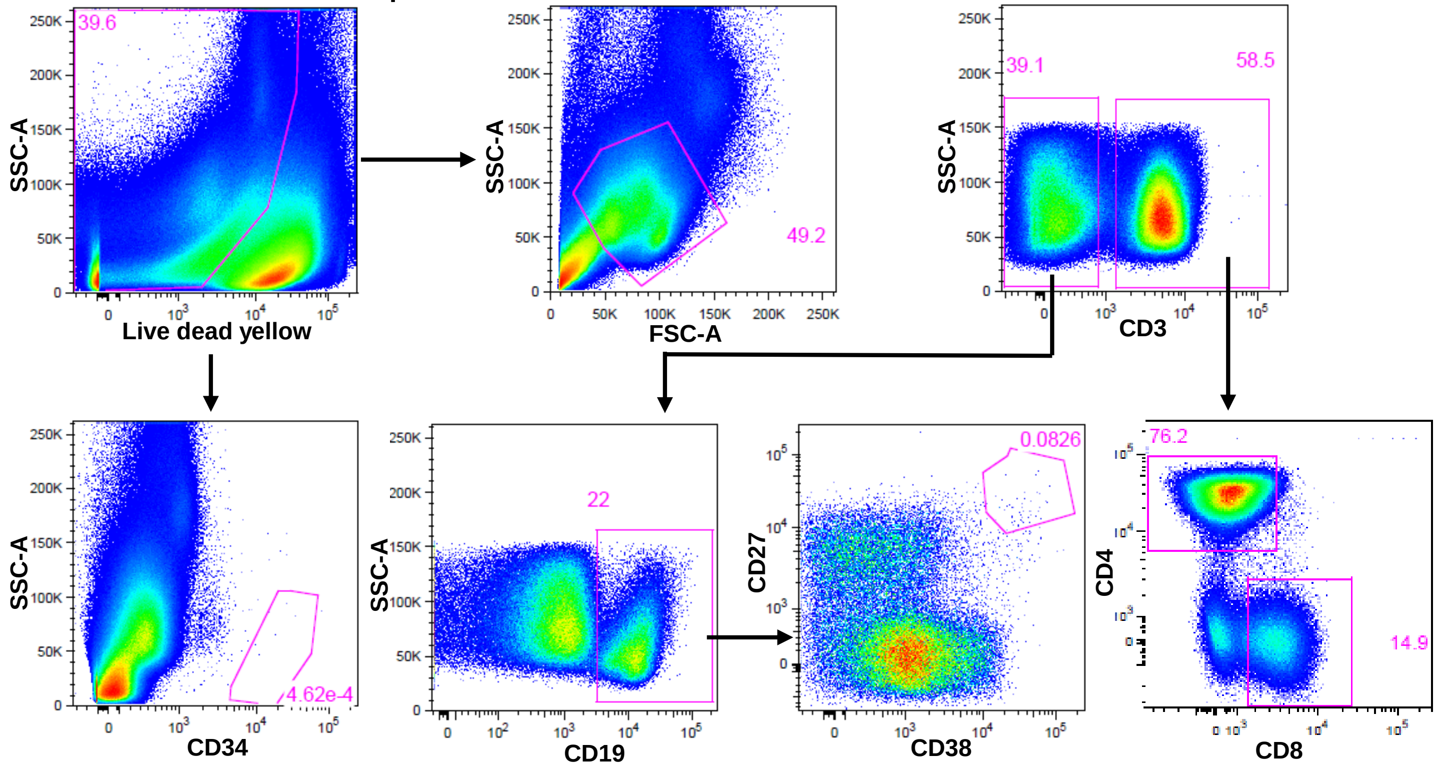

Supplementary Figure 1.3 Basic flow cytometric phenotyping of PBMCs from blood donor #3. In "panel 1", cells in CD27 plots are from the CD3-CD19+ gate. SSC-A, side scatter area; FSC-A, forward scatter area.

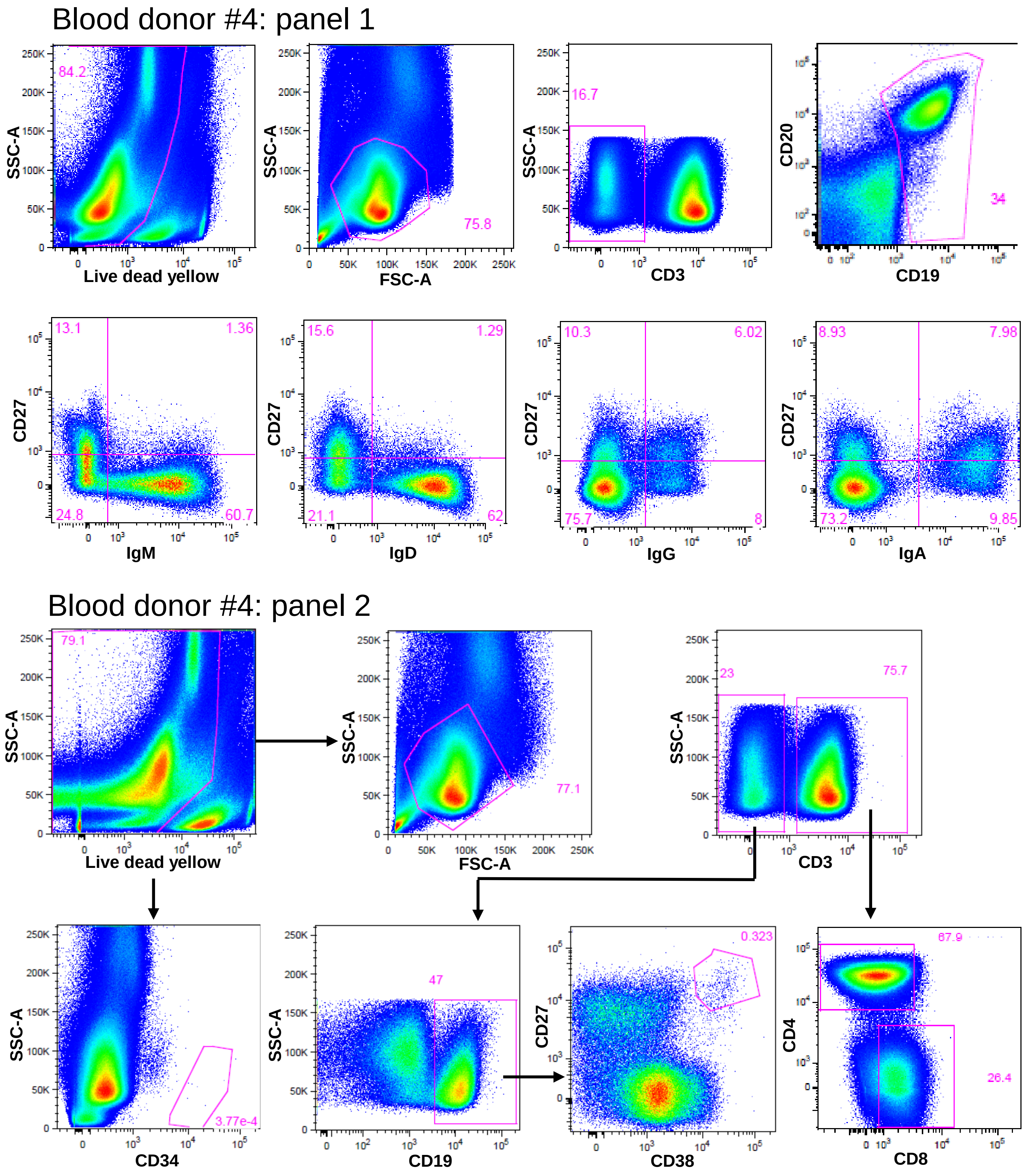

Supplementary Figure 1.4 Basic flow cytometric phenotyping of PBMCs from blood donor #4. In "panel 1", cells in CD27 plots are from the CD3-CD19+ gate. SSC-A, side scatter area; FSC-A, forward scatter area.

Blood donor #5: panel 1

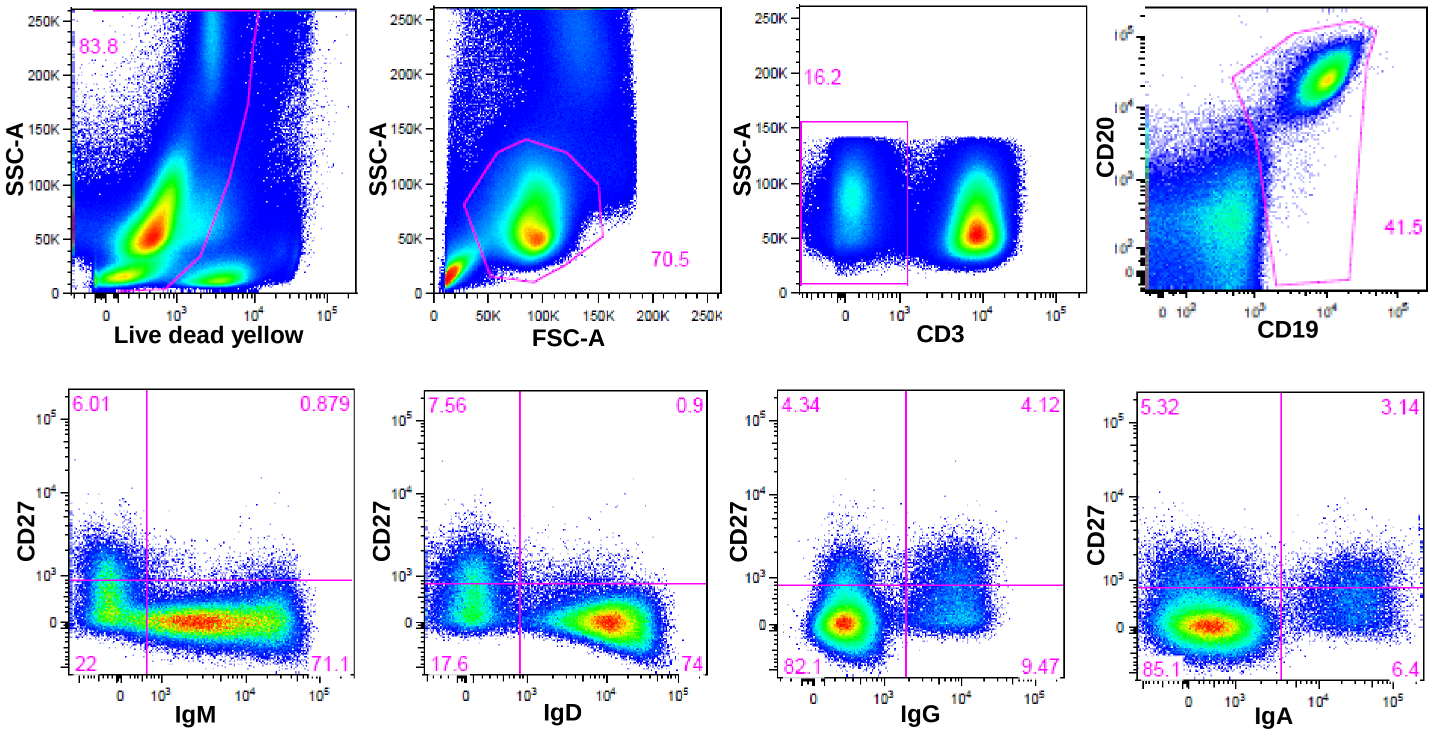

Blood donor #5: panel 2

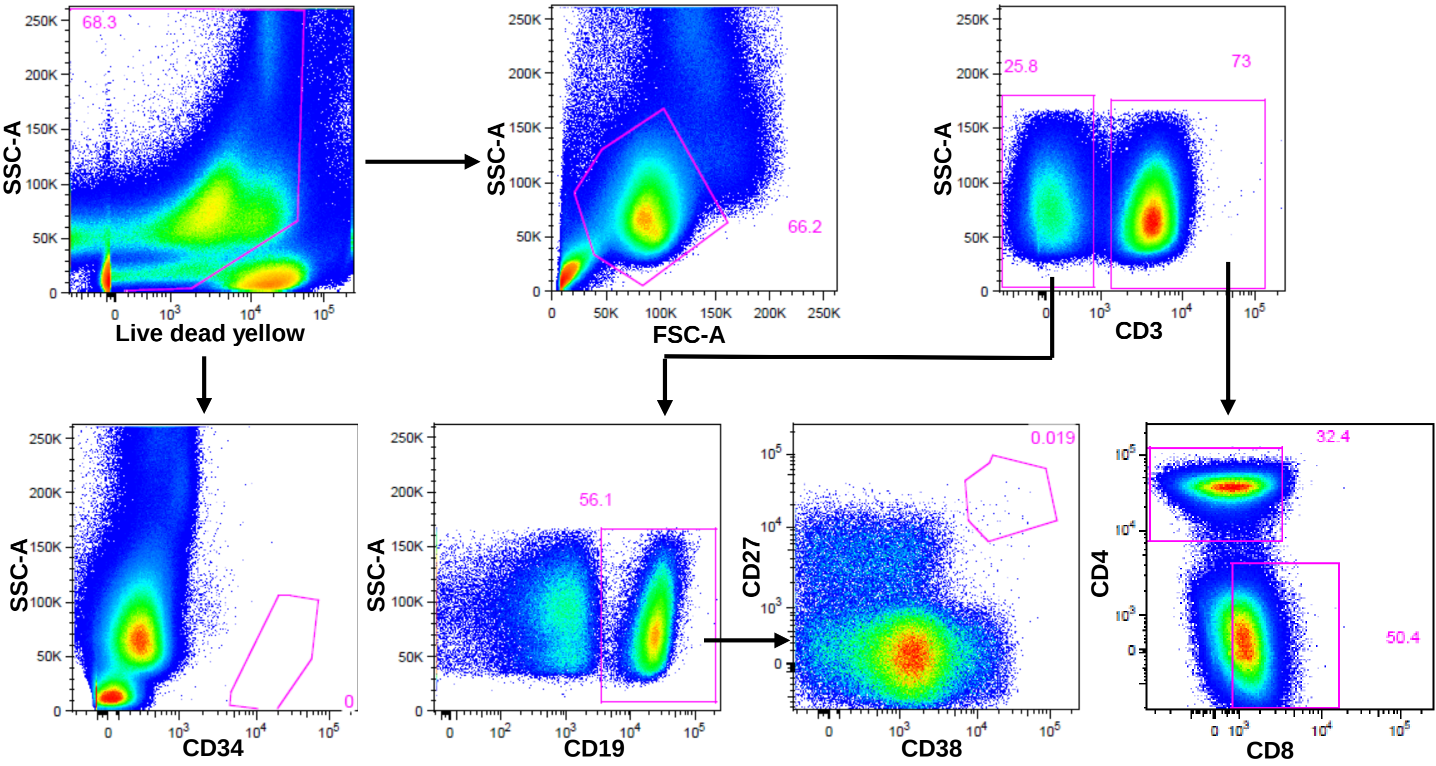

Supplementary Figure 1.5 Basic flow cytometric phenotyping of PBMCs from blood donor #5. In "panel 1", cells in CD27 plots are from the CD3-CD19+ gate. SSC-A, side scatter area; FSC-A, forward scatter area.

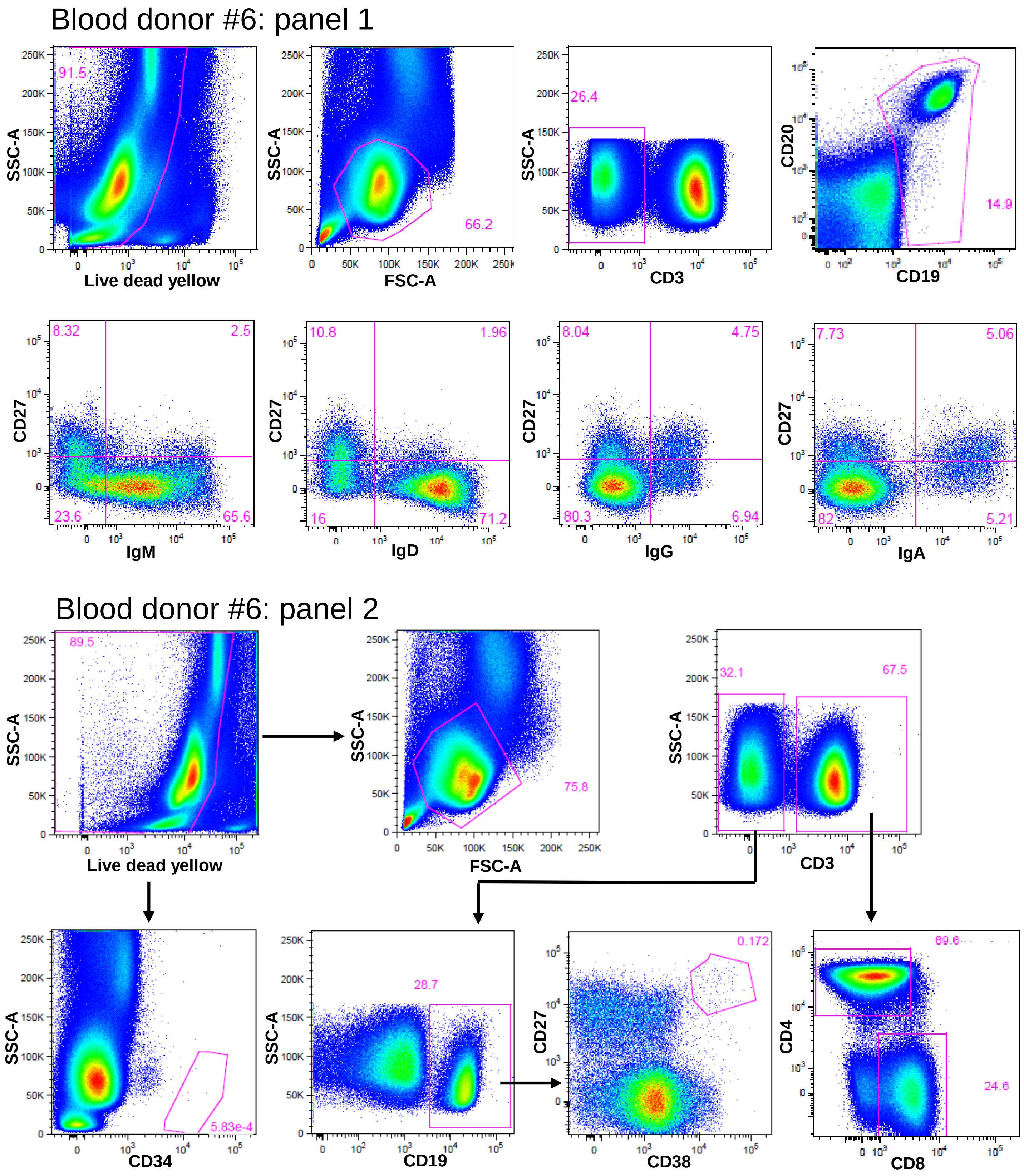

Supplementary Figure 1.6 Basic flow cytometric phenotyping of PBMCs from blood donor #6. In “panel 1”, cells in CD27 plots are from the CD3-CD19+ gate. SSC-A, side scatter area; FSC-A, forward scatter area.

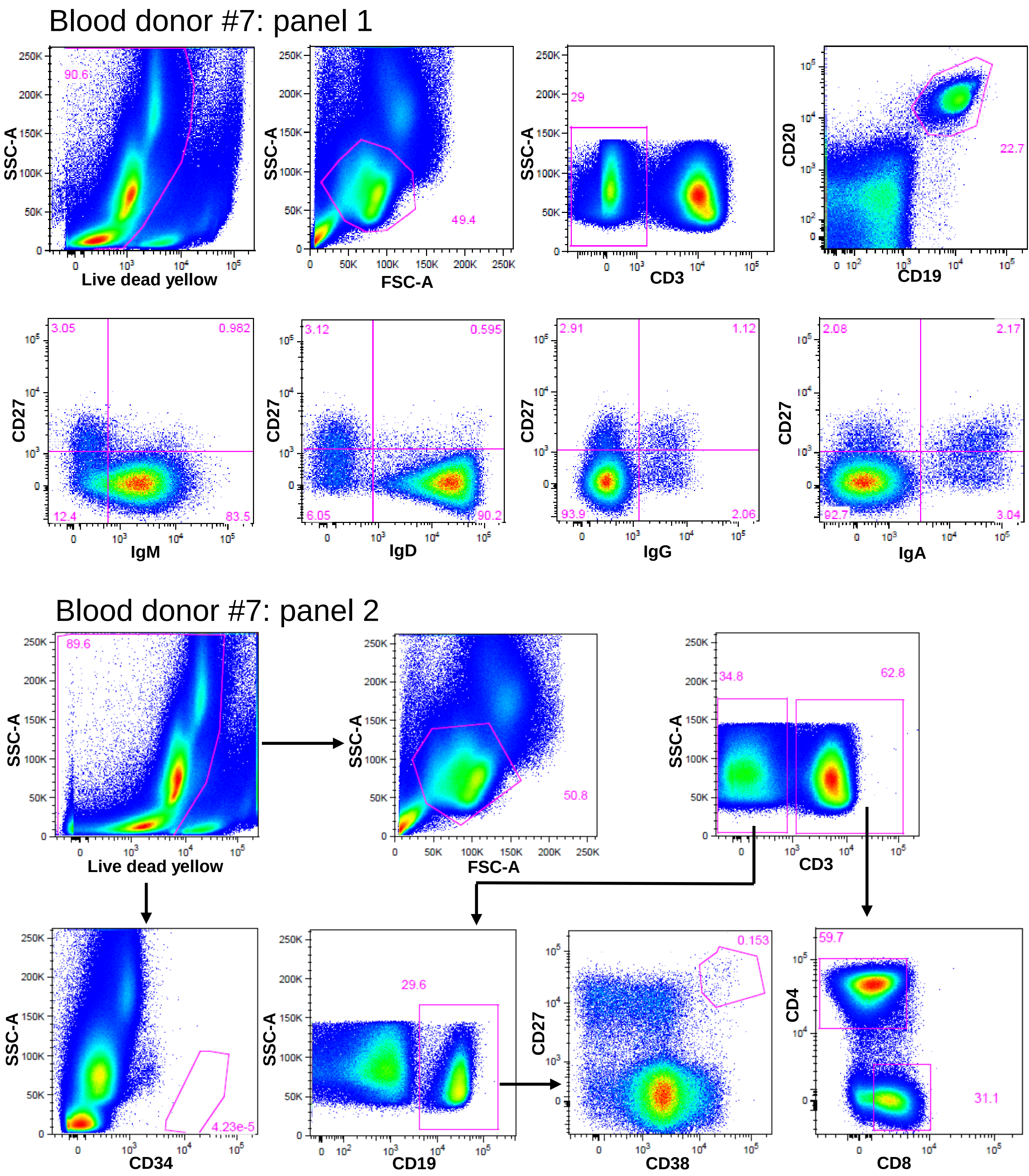

Supplementary Figure 1.7 Basic flow cytometric phenotyping of PBMCs from blood donor #7. In “panel 1”, cells in CD27 plots are from the CD3-CD19+ gate. SSC-A, side scatter area; FSC-A, forward scatter area.

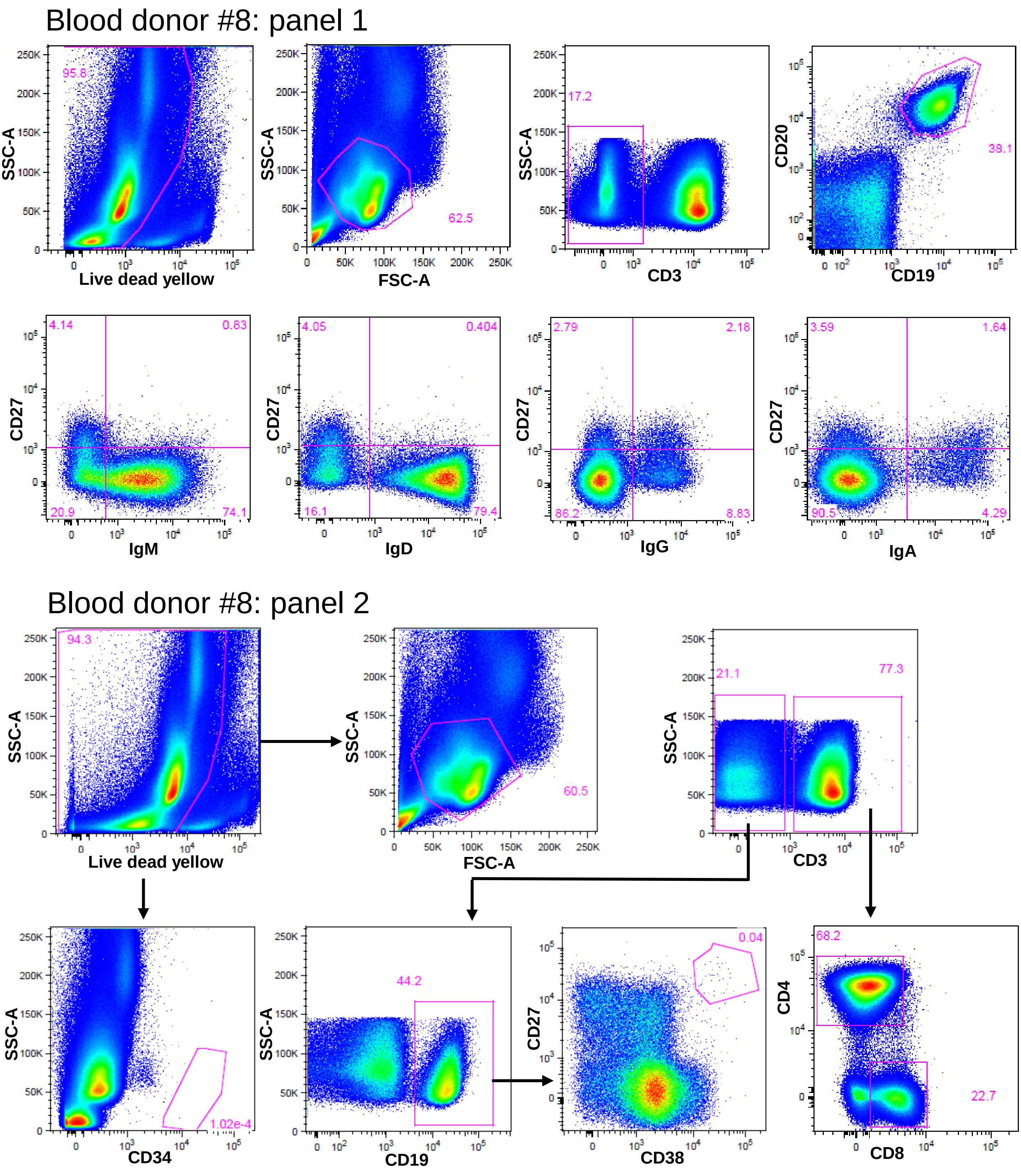

Supplementary Figure 1.8 Basic flow cytometric phenotyping of PBMCs from blood donor #8. In “panel 1”, cells in CD27 plots are from the CD3-CD19+ gate. SSC-A, side scatter area; FSC-A, forward scatter area.

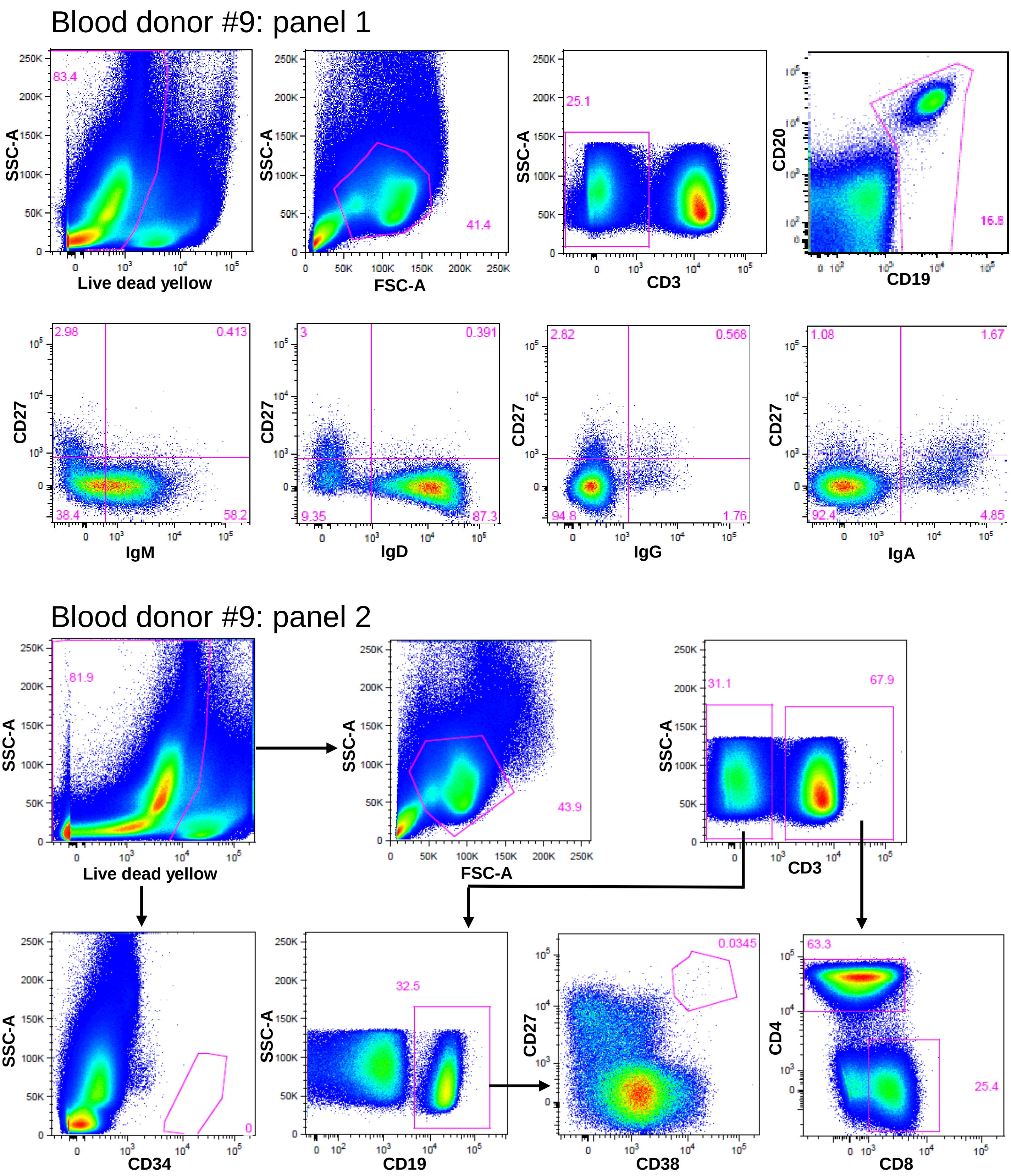

Supplementary Figure 1.9 Basic flow cytometric phenotyping of PBMCs from blood donor #9. In "panel 1", cells in CD27 plots are from the CD3-CD19+ gate. SSC-A, side scatter area; FSC-A, forward scatter area.

Blood donor #10: panel 1

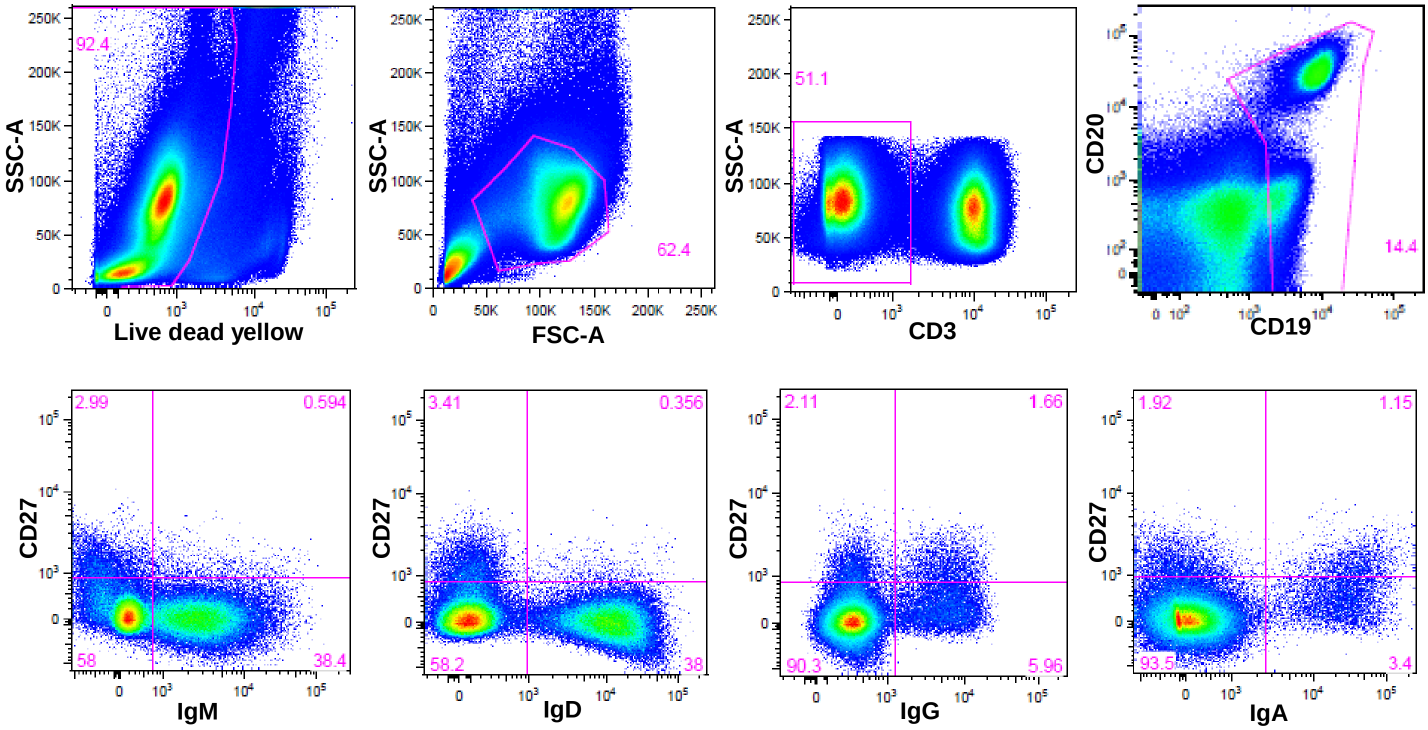

Blood donor #10: panel 2

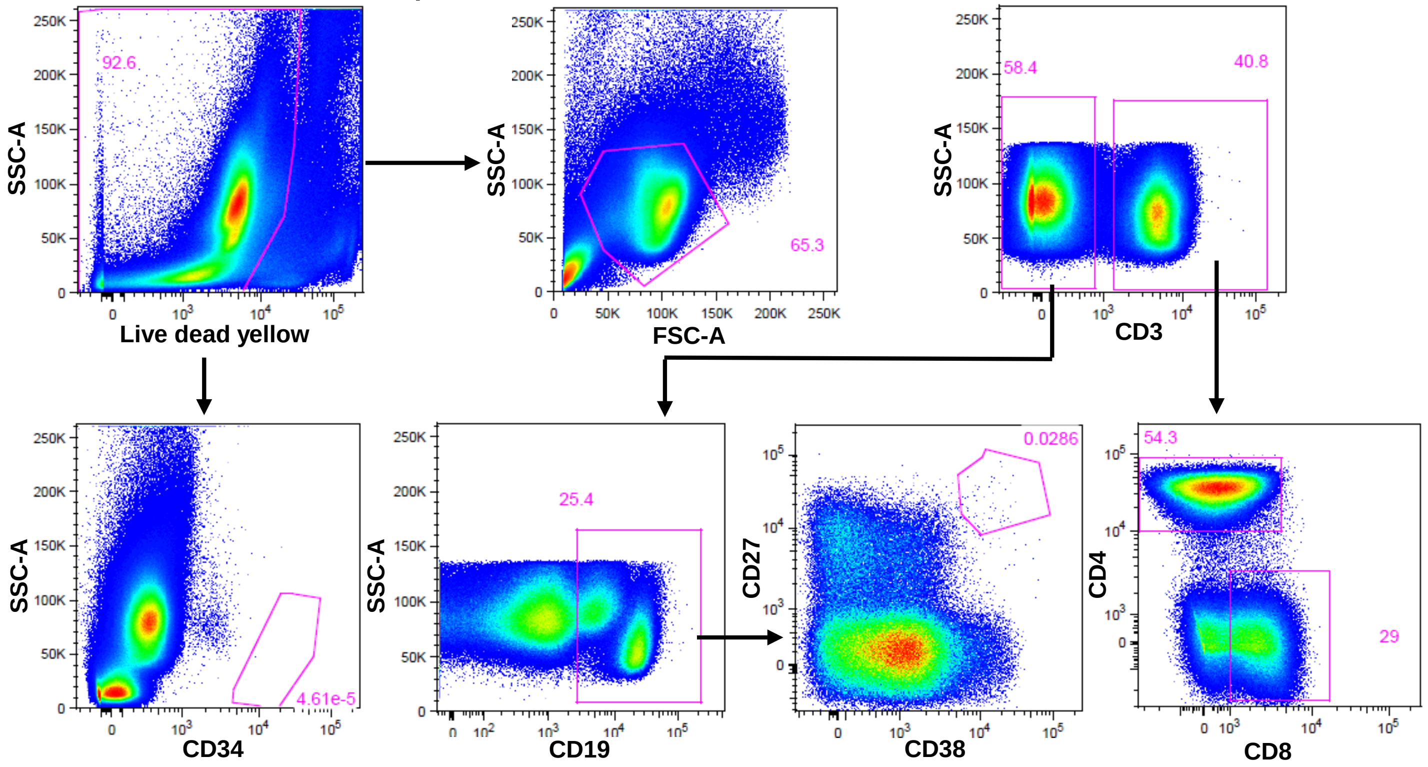

Supplementary Figure 1.10 Basic flow cytometric phenotyping of PBMCs from blood donor #10. In “panel 1”, cells in CD27 plots are from the CD3-CD19+ gate. SSC-A, side scatter area; FSC-A, forward scatter area.

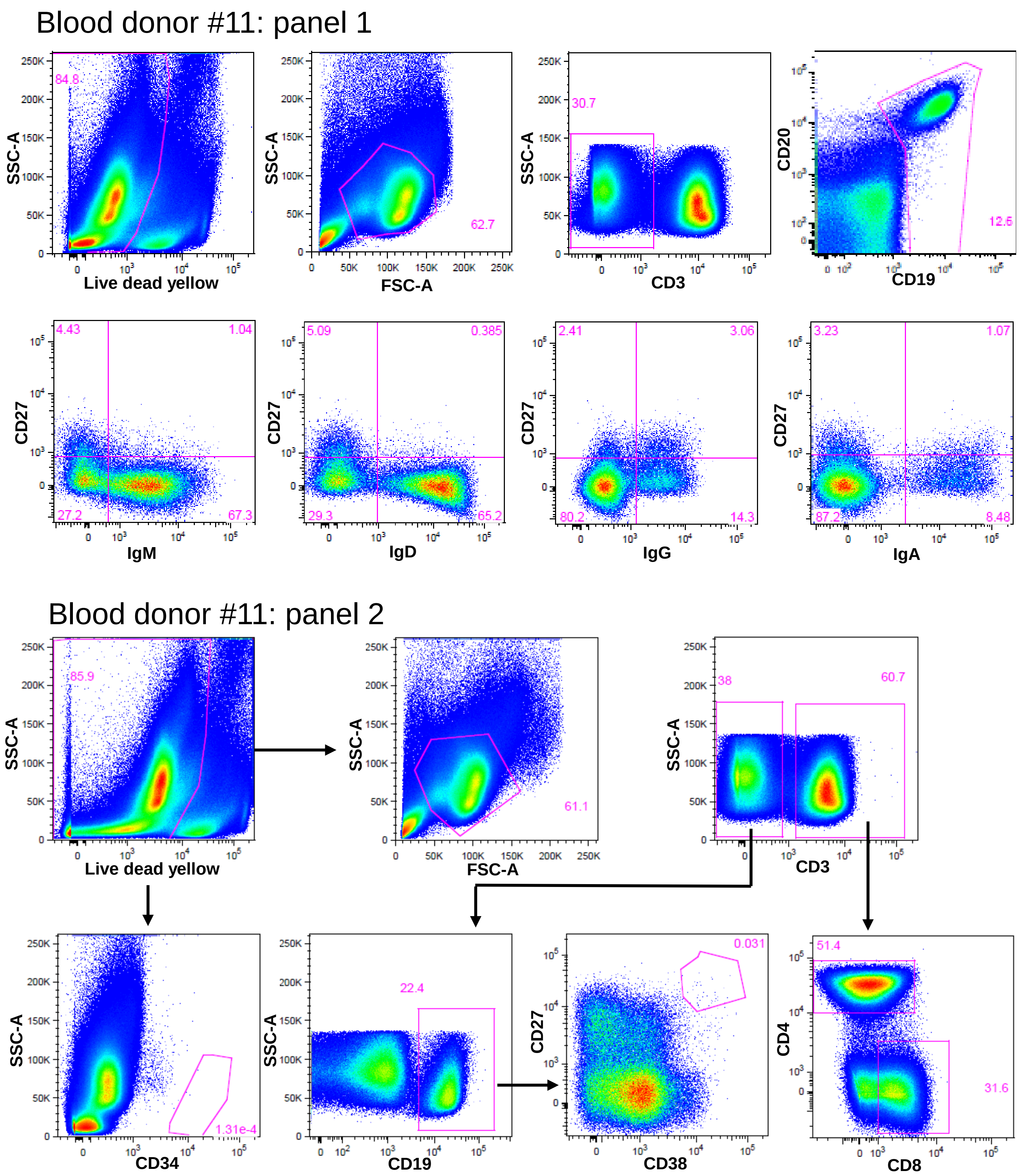

Supplementary Figure 1.11 Basic flow cytometric phenotyping of PBMCs from blood donor #11. In "panel 1", cells in CD27 plots are from the CD3-CD19+ gate. SSC-A, side scatter area; FSC-A, forward scatter area.

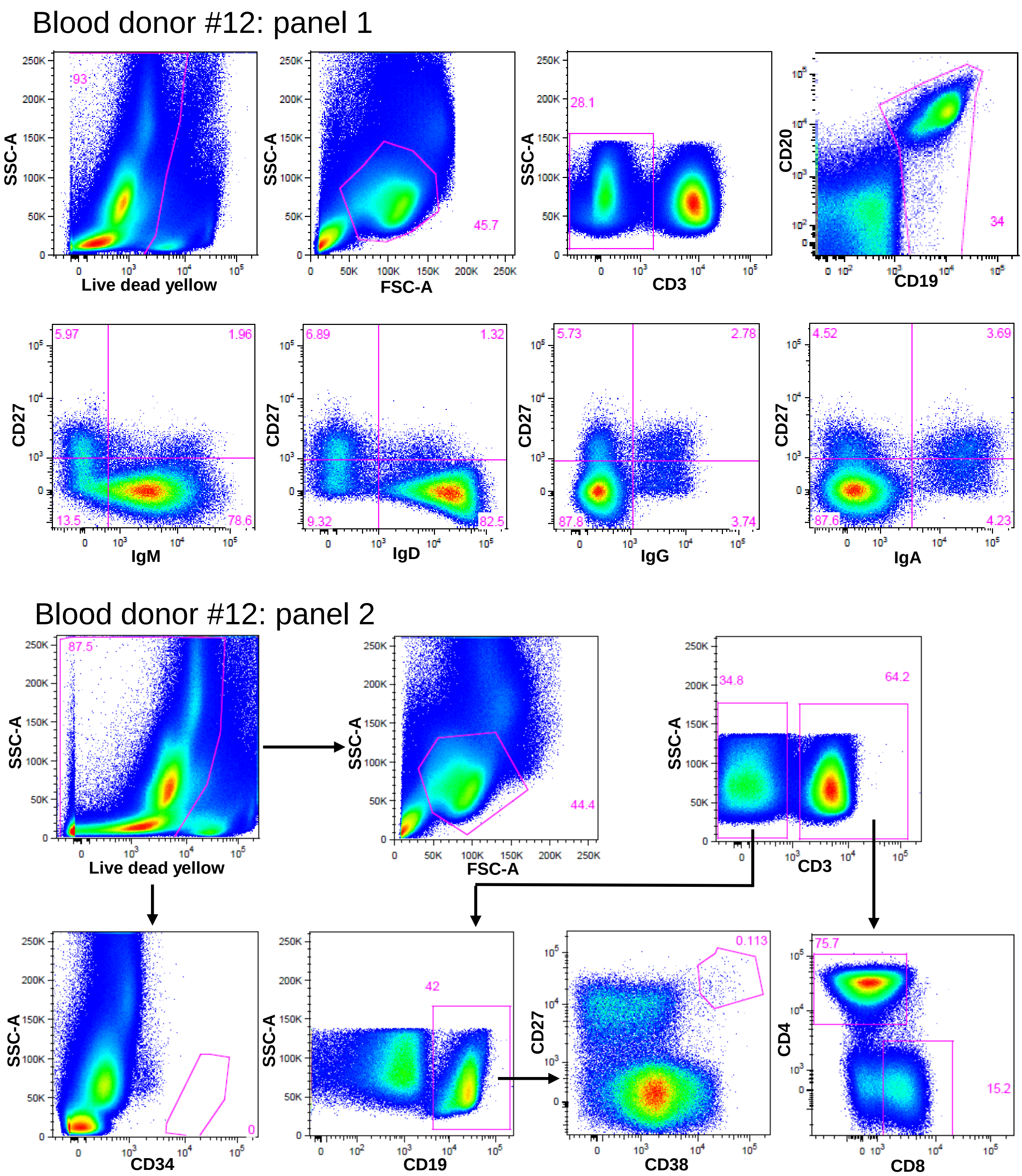

Supplementary Figure 1.12 Basic flow cytometric phenotyping of PBMCs from blood donor #12. In "panel 1", cells in CD27 plots are from the CD3-CD19+ gate. SSC-A, side scatter area; FSC-A, forward scatter area.

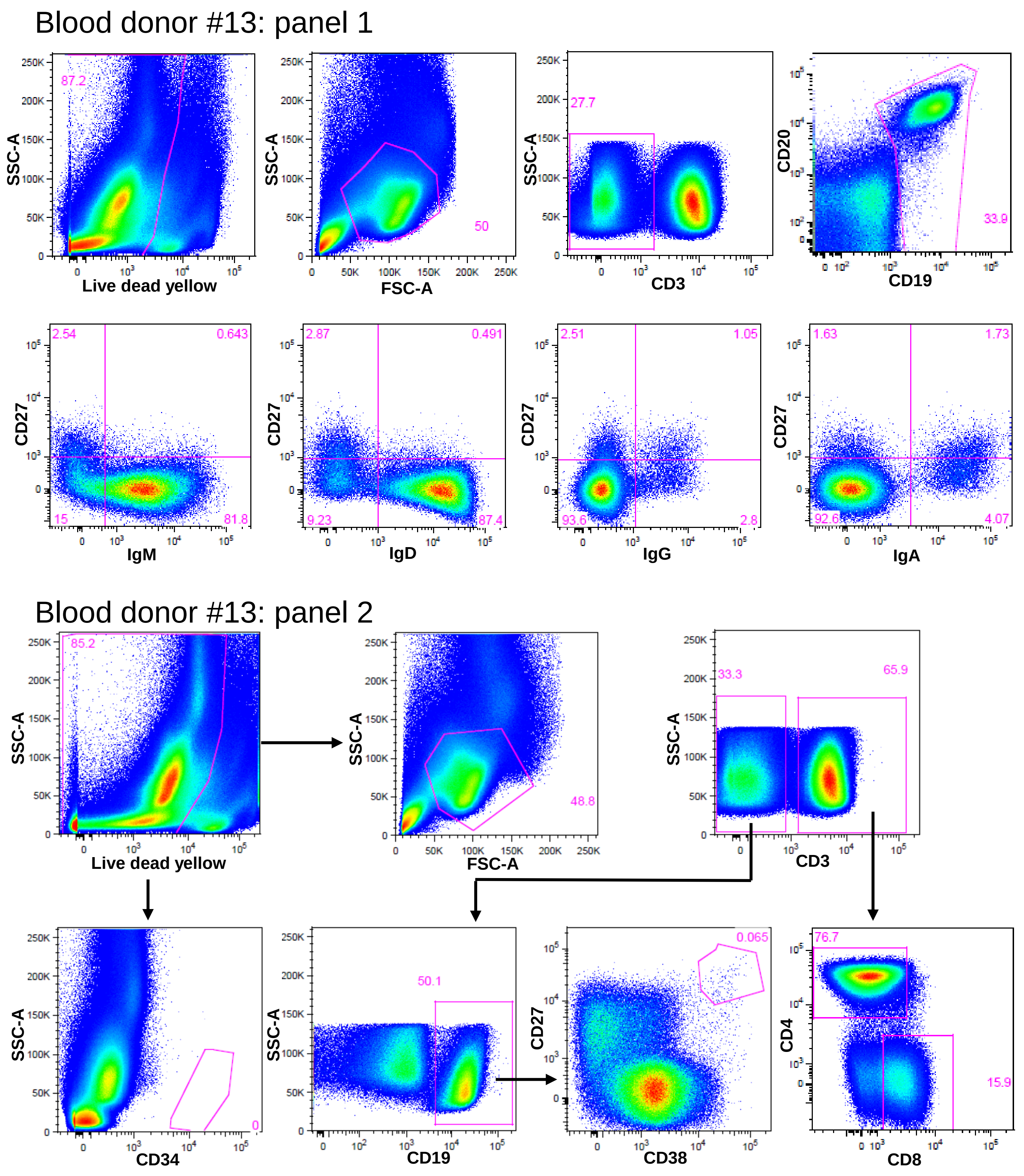

Supplementary Figure 1.13 Basic flow cytometric phenotyping of PBMCs from blood donor #13. In “panel 1”, cells in CD27 plots are from the CD3-CD19+ gate. SSC-A, side scatter area; FSC-A, forward scatter area.

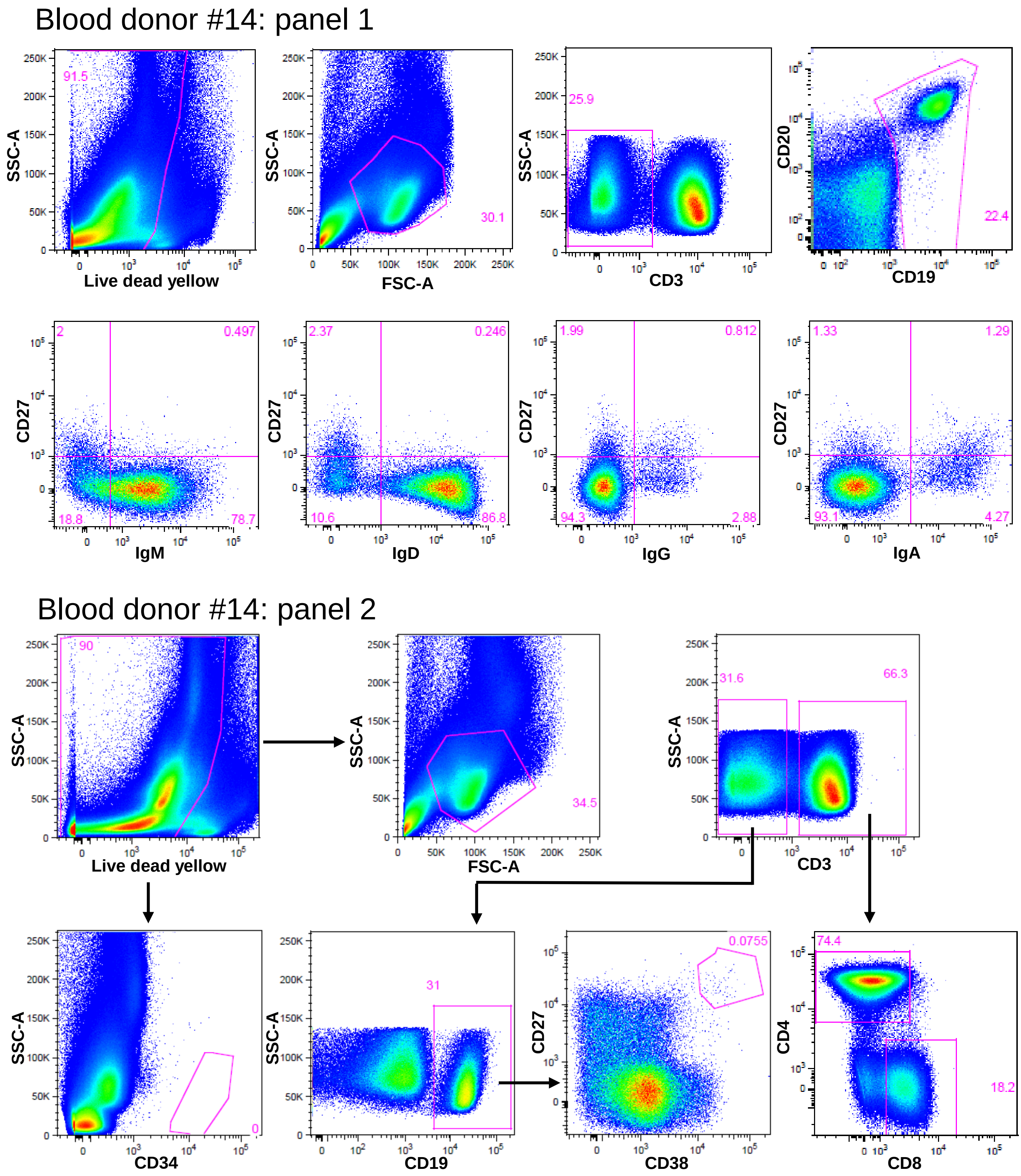

Supplementary Figure 1.14 Basic flow cytometric phenotyping of PBMCs from blood donor #14. In "panel 1", cells in CD27 plots are from the CD3-CD19+ gate. SSC-A, side scatter area; FSC-A, forward scatter area.

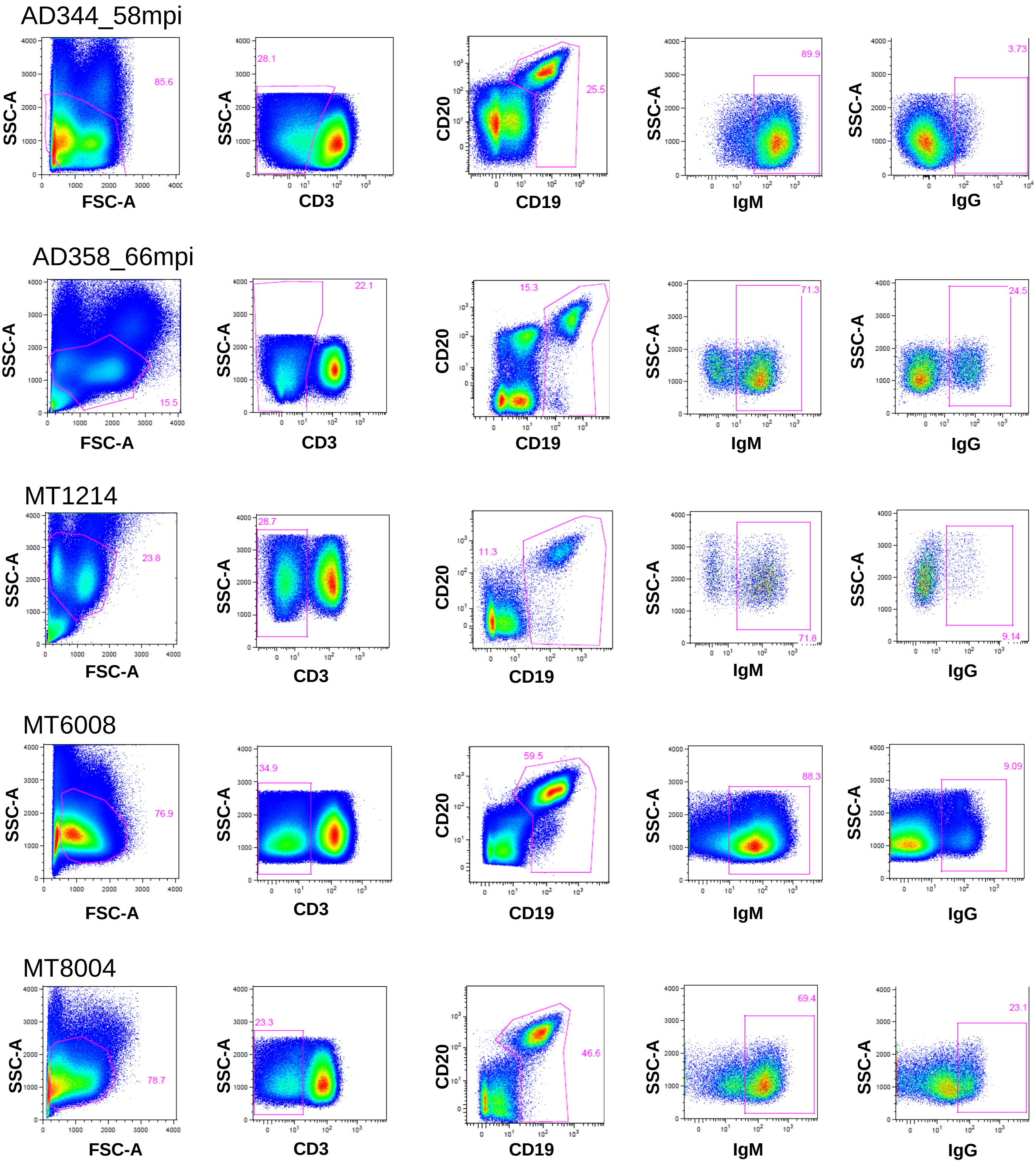

Supplementary Figure 1.15 Basic flow cytometric phenotyping of PBMCs from five HIV-1 infected individuals. The IgM and IgG plots are independently from the CD3-CD19+ gate. SSC-A, side scatter area; FSC-A, forward scatter area.

Cord blood #1: panel 1

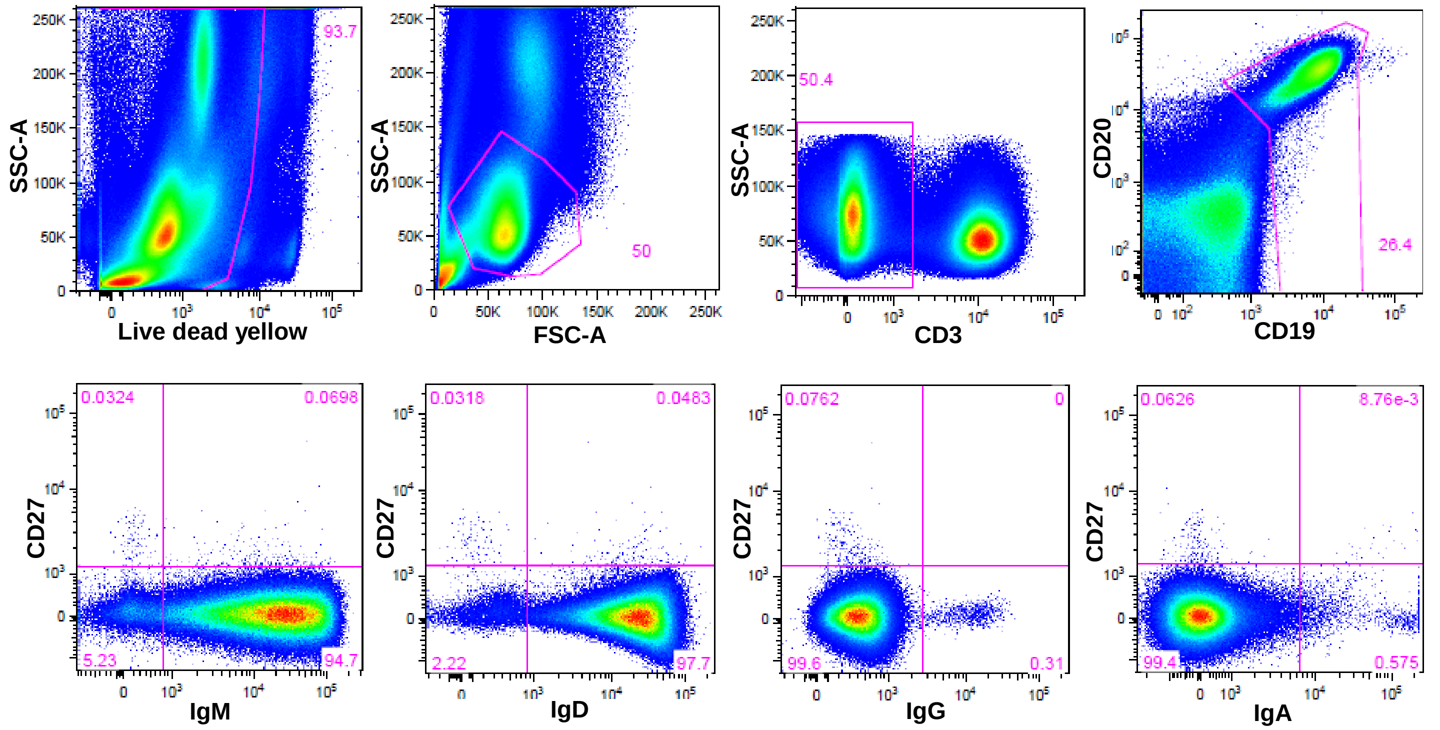

Cord blood #1: panel 2

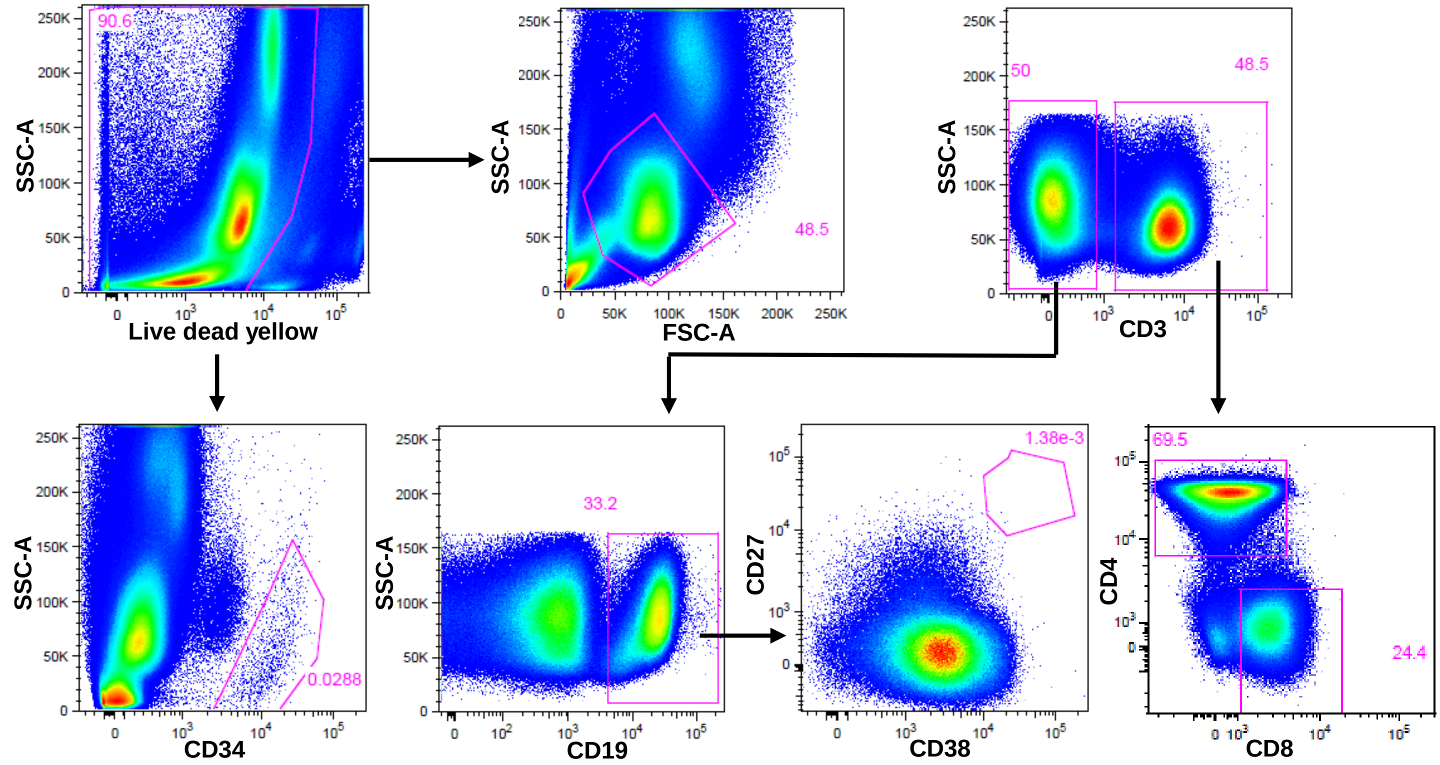

Supplementary Figure 1.16 Basic flow cytometric phenotyping of cord blood #1. In “panel 1”, cells in CD27 plots are from the CD3-CD19+ gate. SSC-A, side scatter area; FSC-A, forward scatter area.

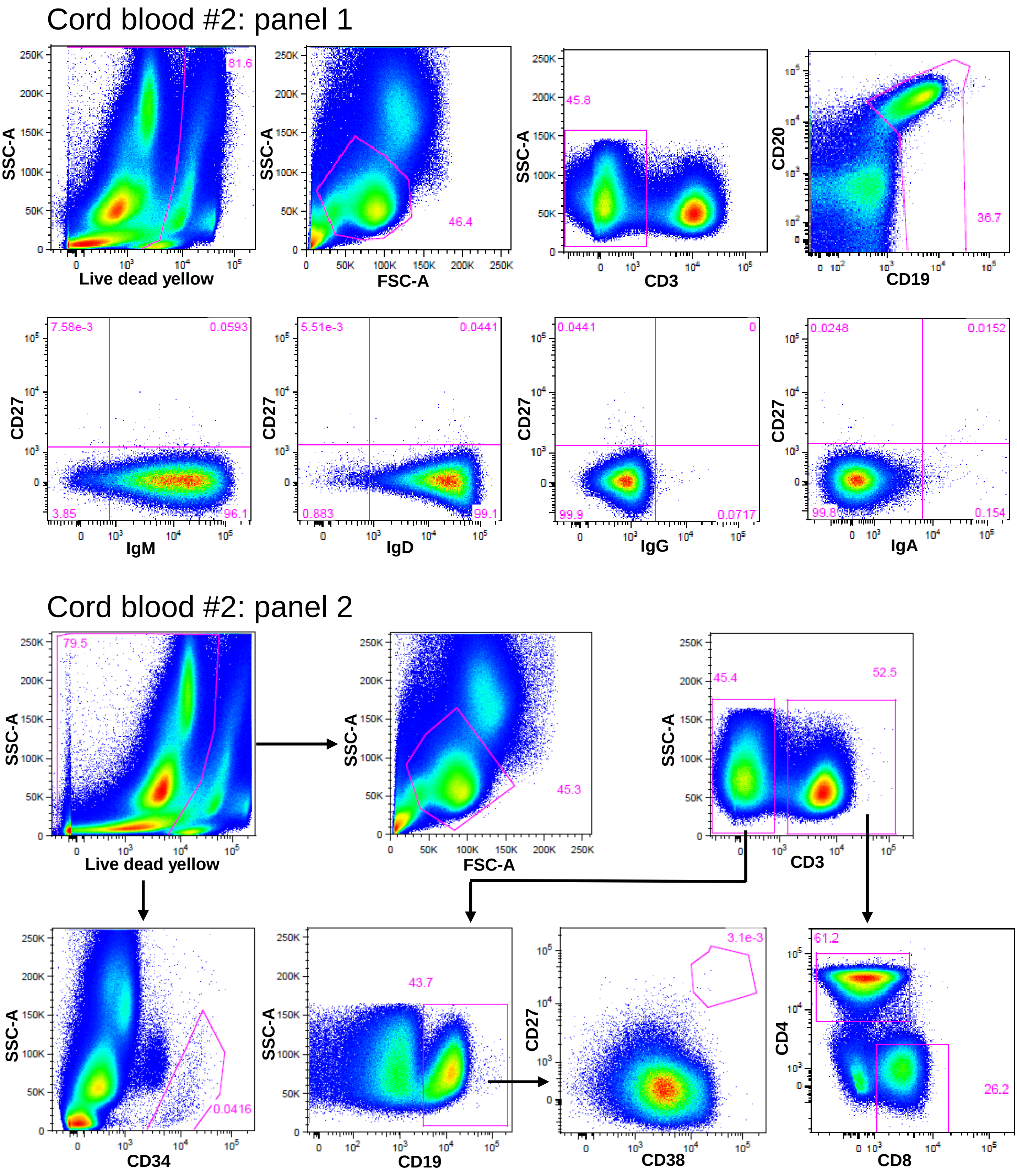

Supplementary Figure 1.17 Basic flow cytometric phenotyping of cord blood #2. In “panel 1”, cells in CD27 plots are from the CD3-CD19+ gate. SSC-A, side scatter area; FSC-A, forward scatter area.

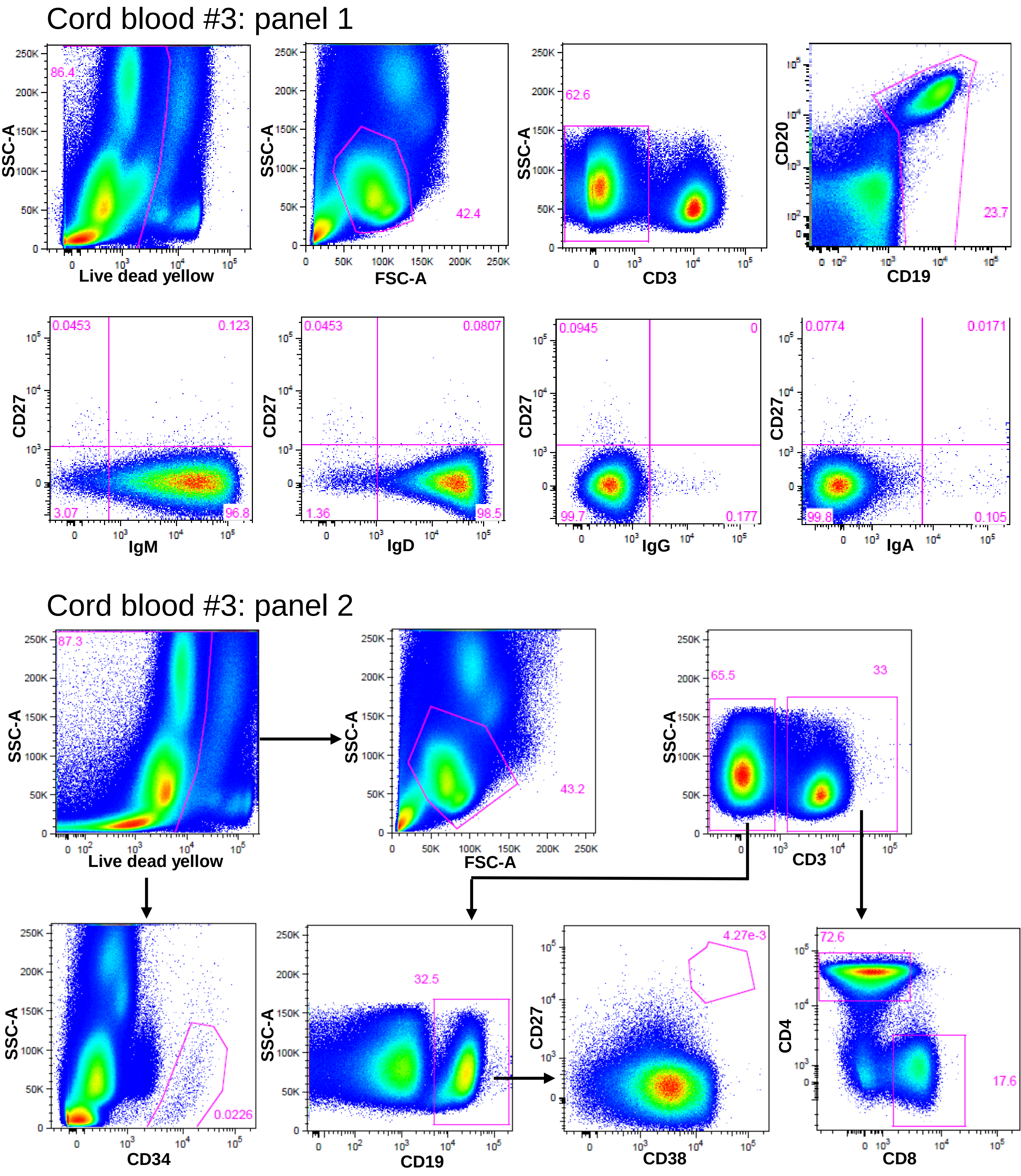

Supplementary Figure 1.18 Basic flow cytometric phenotyping of cord blood #3. In “panel 1”, cells in CD27 plots are from the CD3-CD19+ gate. SSC-A, side scatter area; FSC-A, forward scatter area.

Cord blood #4: panel 1

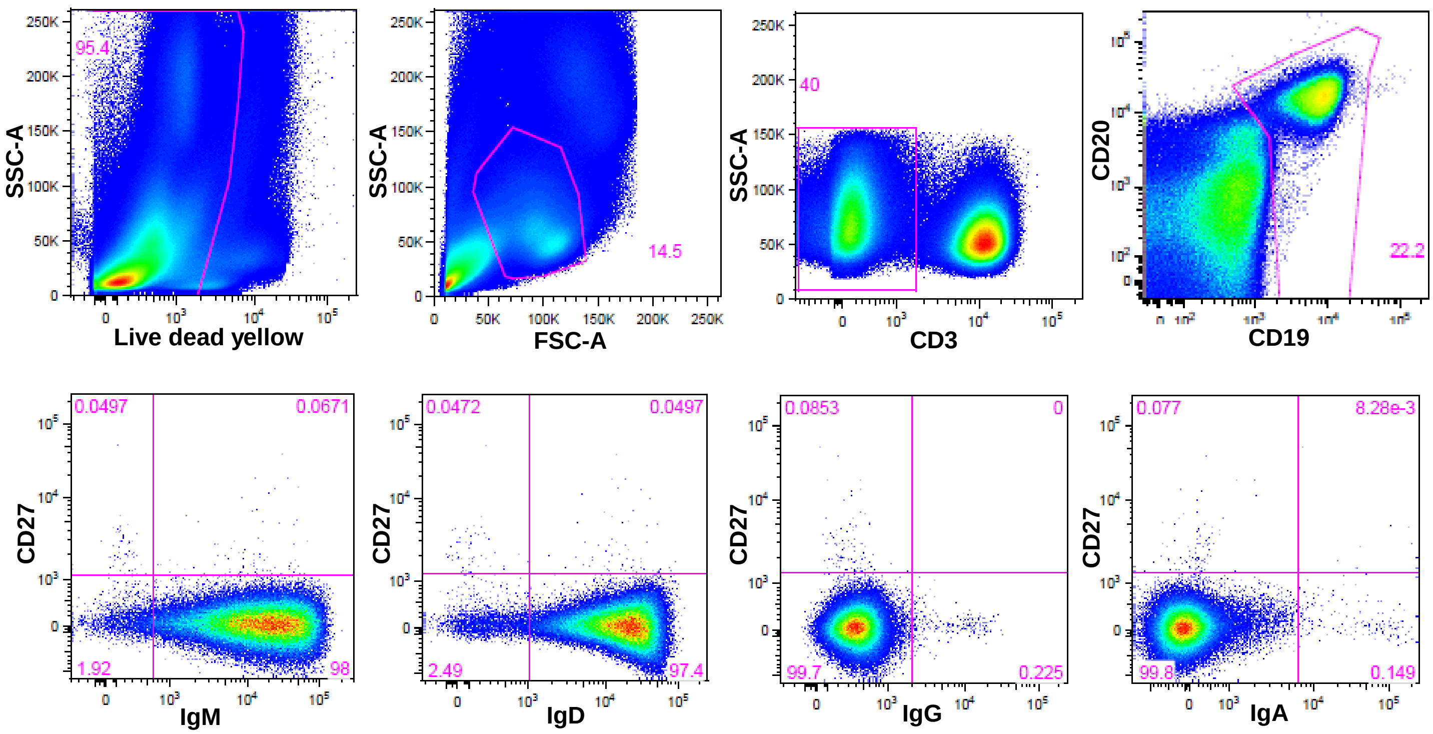

Cord blood #4: panel 2

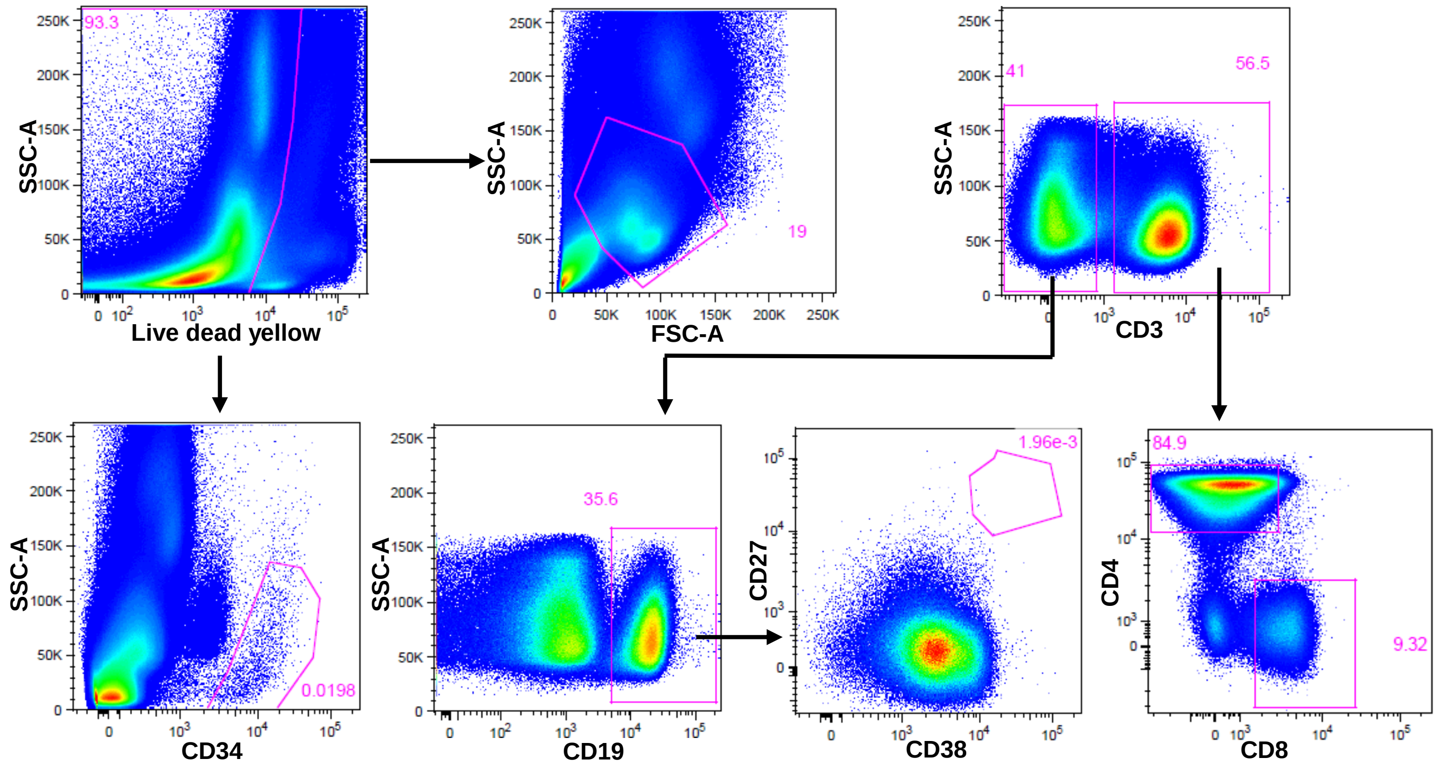

Supplementary Figure 1.19 Basic flow cytometric phenotyping of cord blood #4. In "panel 1", cells in CD27 plots are from the CD3-CD19+ gate. SSC-A, side scatter area; FSC-A, forward scatter area.

Cord blood #5: panel 1

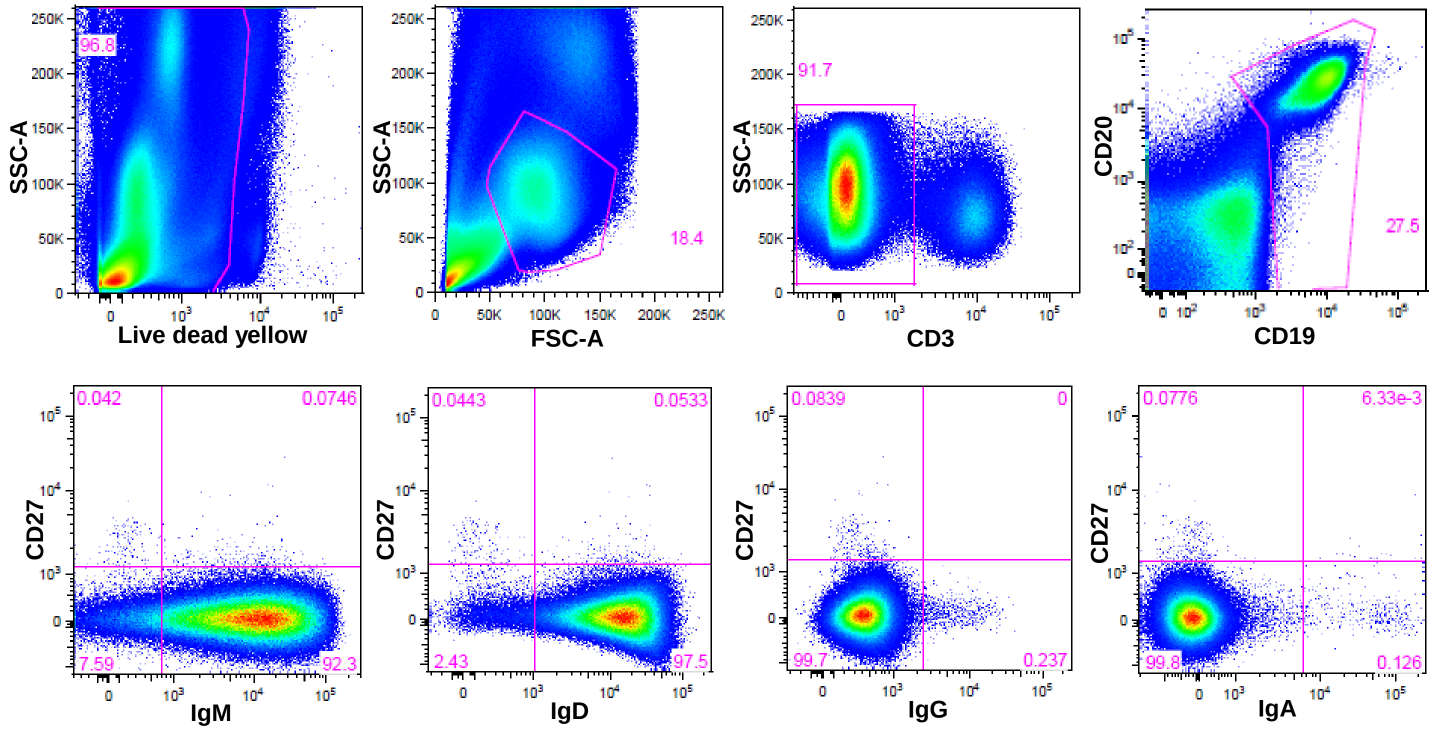

Cord blood #5: panel 2

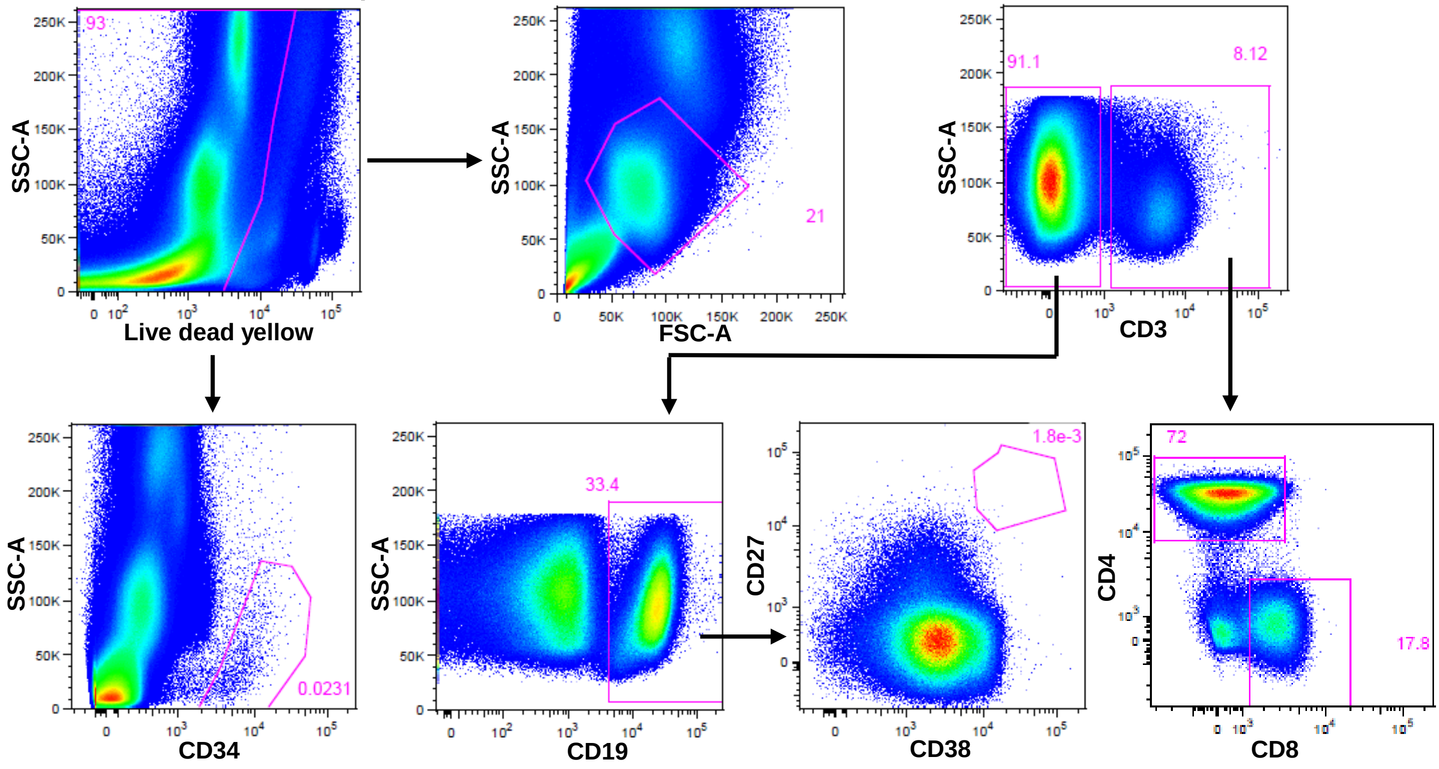

Supplementary Figure 1.20 Basic flow cytometric phenotyping of cord blood #5. In "panel 1", cells in CD27 plots are from the CD3-CD19+ gate. SSC-A, side scatter area; FSC-A, forward scatter area.

HIS-CD4/B mouse #770 (naïve)

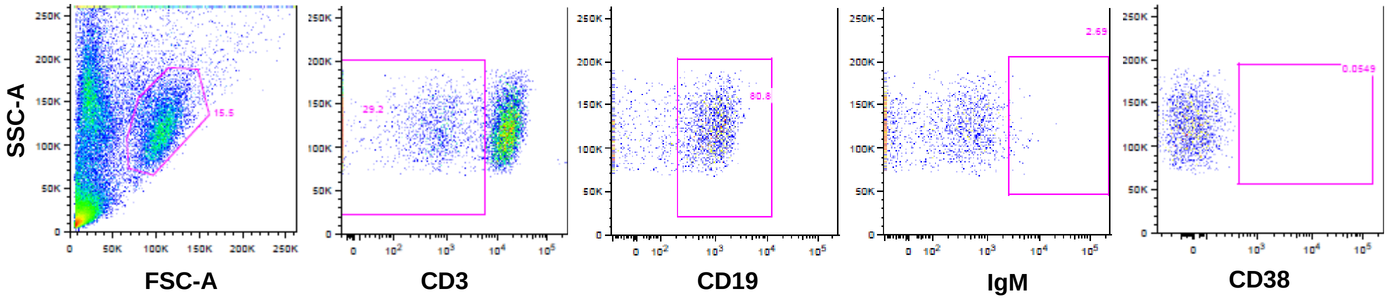

HIS-CD4/B mouse #771 (injected with sheep red blood cells)

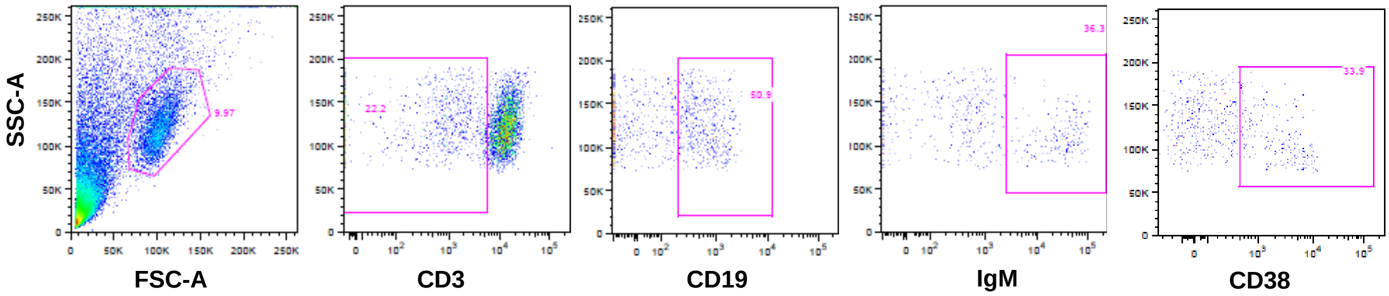

HIS-CD4/B mouse #755 (immunized with a malaria protein)

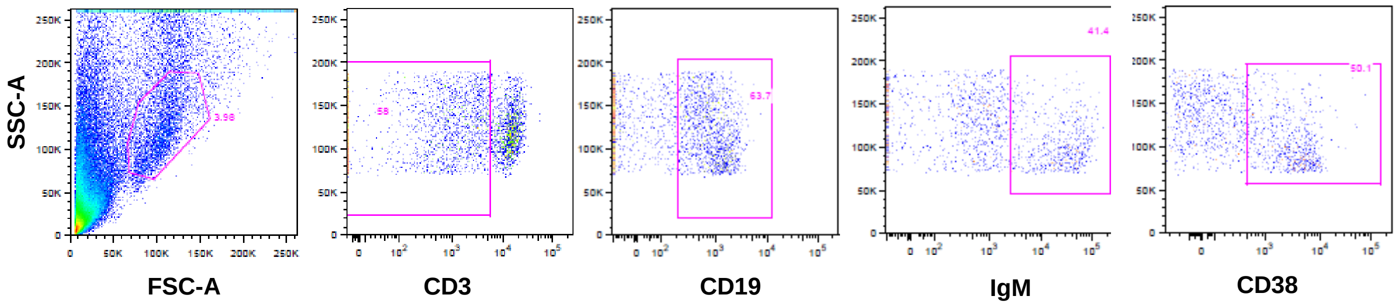

Supplementary Figure 1.21 Basic flow cytometric phenotyping of human B cells in splenocytes of three HIS-CD4/B mice from 26 weeks after human stem cell engraftment. The IgM and CD38 plots are independently from the CD3-CD19+ gate. SSC-A, side scatter area; FSC-A, forward scatter area.

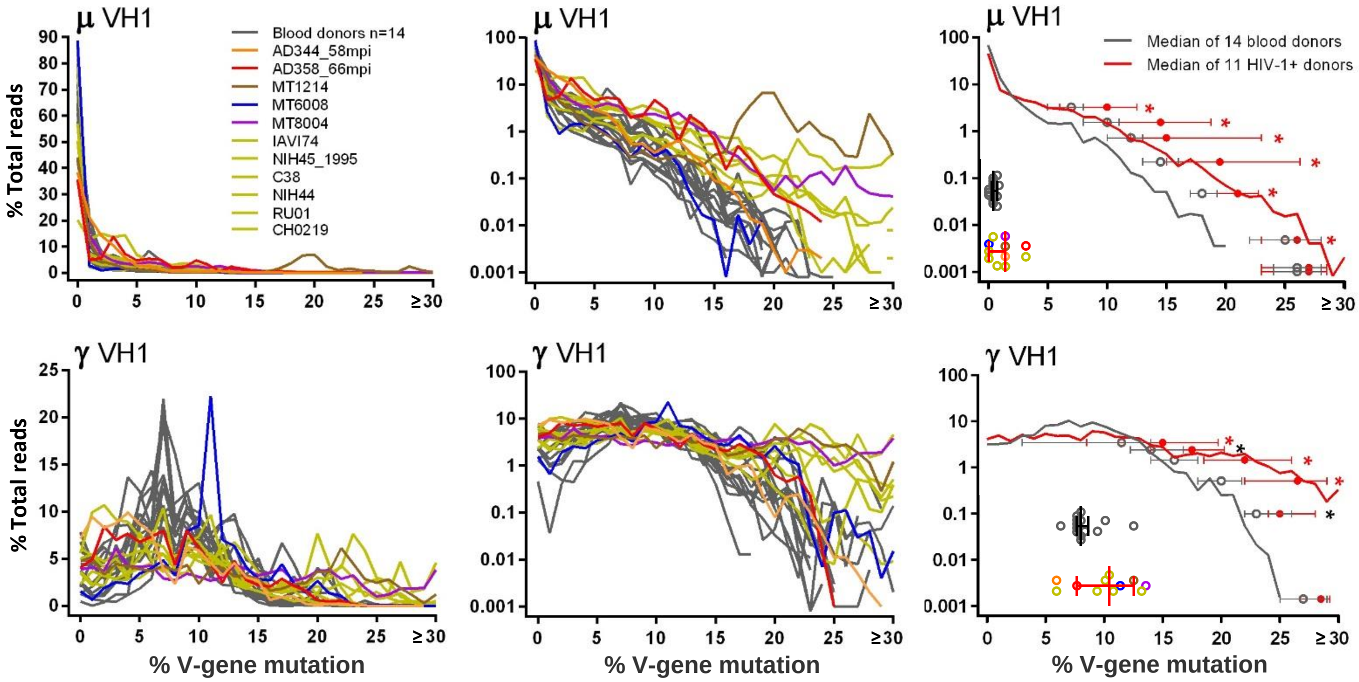

Supplementary Figure 2. Comparison of VH1 mutation frequencies in 14 blood donor controls versus 11 HIV-1 infected individuals, including previously published 454 data from 6 HIV-1+ individuals. In left and middle panels, y-axis shows the % of total reads (linear scale on left and log scale in middle) with indicated levels of VH1 mutations on x-axis. Gray lines indicate blood donors; colored lines indicate HIV-1+ donors, with the published 454 data colored lime. Right panels indicate comparisons of median mutation values (scatter plotted gray circles for blood donors and color circles for HIV-1+ donors, with median and interquartile ranges) and median distribution curves (gray for blood donors and red for HIV-1+ donors), with median and interquartile ranges indicated for each decile bin of % total reads where the two groups separate. Asterisks indicate significant differences, with red asterisks indicating significance based on FDR adjusted P values.

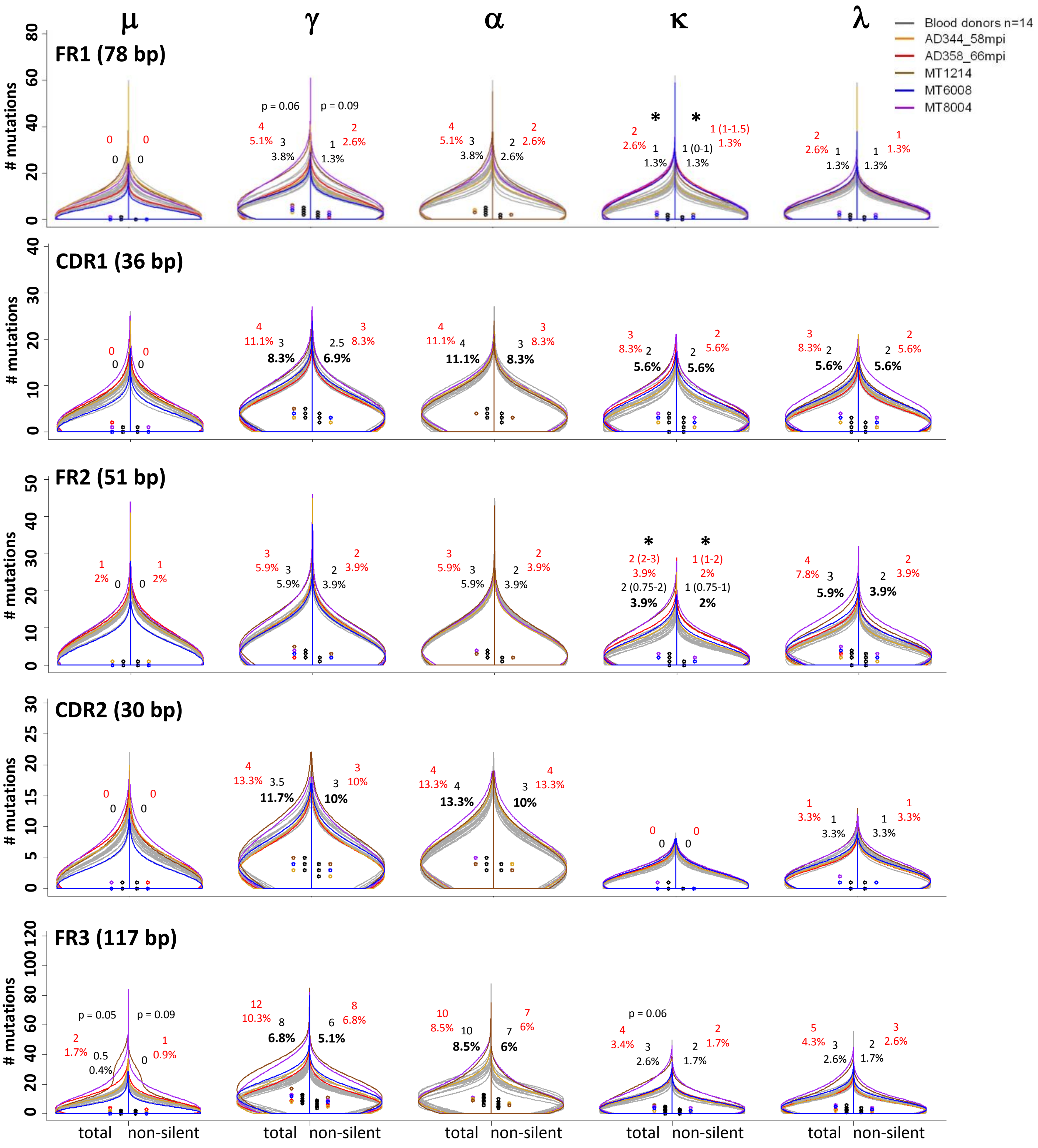

Supplementary Figure 3. Violin plot of mutation numbers by IMGT-defined V-gene regions for μ, γ, α, κ and λ. For each violin plot, width is proportional to total reads containing the numbers of mutations indicated by height; left side indicates total mutations, and right side indicates non-silent mutations. Gray lines indicate blood donors, and colored lines indicate HIV-1+ donors, with scattered dots inside the violin indicating individual median mutation numbers. Numbers outside of violin indicate group medians (black for blood donors and red for HIV-1+), along with mutations in percentage. Asterisks indicate significant differences between the two groups; for differences with identical group medians, the interquartile ranges are shown. For each chain type other than μ, more mutated regions are highlighted with bold median % mutations.
